# Evaluating Three Modelling Frameworks for Assessing Changes in Fin Whale Distribution in the Mediterranean Sea

**DOI:** 10.1002/ece3.71007

**Published:** 2025-03-07

**Authors:** Francesca Grossi, Elliott L. Hazen, Giulio De Leo, Léa David, Nathalie Di‐Méglio, Antonella Arcangeli, Eugenia Pasanisi, Ilaria Campana, Miriam Paraboschi, Alberto Castelli, Massimiliano Rosso, Aurelie Moulins, Paola Tepsich

**Affiliations:** ^1^ CIMA Research Foundation Savona Italy; ^2^ DIBRIS University of Genoa Genova Italy; ^3^ Ecosystem Science Division Southwest Fisheries Science Center Monterey California USA; ^4^ Institute of Marine Science University of California Santa Cruz Santa Cruz California USA; ^5^ Hopkins Marine Station, Department of Biology Stanford University Pacific Grove California USA; ^6^ Department of Earth System Science Stanford University Stanford California USA; ^7^ EcoOcéan Institut Montpellier France; ^8^ Department for Biodiversity Conservation and Monitoring ISPRA Rome Italy; ^9^ Department of Environmental Biology Sapienza University of Rome Rome Italy; ^10^ Accademia del Leviatano Maccarese Italy; ^11^ University of Pisa Pisa Italy; ^12^ National Biodiversity Future Center (NBFC) Palermo Italy

**Keywords:** BRT, fin whale, GAM, habitat model, hurdle model, Mediterranean Sea

## Abstract

Understanding the habitat of highly migratory species is aided by using species distribution models to identify species‐habitat relationships and to inform conservation and management plans. While Generalized Additive Models (GAMs) are commonly used in ecology, and particularly the habitat modeling of marine mammals, there remains a debate between modeling habitat (presence/absence) versus density (# individuals). Our study assesses the performance and predictive capabilities of GAMs compared to boosted regression trees (BRTs) for modeling both fin whale density and habitat suitability alongside Hurdle Models treating presence/absence and density as a two‐stage process to address the challenge of zero‐inflated data. Fin whale data were collected from 2008 to 2022 along fixed transects crossing the NW Mediterranean Sea during the summer period. Data were analyzed using traditional line transect methodology, obtaining the Effective Area monitored. Based on existing literature, we select various covariates, either static in nature, such as bathymetry and slope, or variable in time, for example, SST, MLD, Chl concentration, EKE, and FSLE. We compared both the explanatory power and predictive skill of the different modeling techniques. Our results show that all models performed well in distinguishing presences and absences but, while density and presence patterns for the fin whale were similar, their dependencies on environmental factors can vary depending on the chosen model. Bathymetry was the most important variable in all models, followed by SST and the chlorophyll recorded 2 months before the sighting. This study underscores the role SDMs can play in marine mammal conservation efforts and emphasizes the importance of selecting appropriate modeling techniques. It also quantifies the relationship between environmental variables and fin whale distribution in an understudied area, providing a solid foundation for informed decision making and spatial management.

## Graphical abstract

This study evaluates the effectiveness of Generalized Additive Models (GAMs), Boosted Regression Trees (BRTs), and Hurdle Models for modeling fin whale density and habitat suitability in the NW Mediterranean Sea. Key findings reveal that while all models effectively distinguished whale presence and absence, density and habitat relationships varied across models, with bathymetry, SST, and chlorophyll levels being crucial predictors. These insights support marine conservation efforts by refining species distribution modeling techniques.

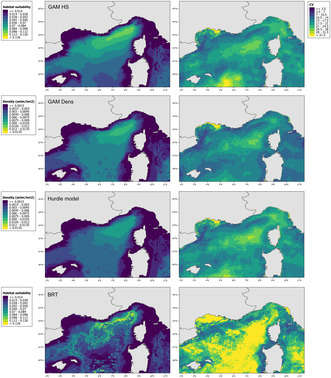

## 1 Introduction

Species distribution models (SDMs) are a useful tool for informing conservation and management strategies. They are now widely used to predict and describe the distribution of highly mobile species such as cetaceans for both conservation and management purposes (Grossi et al. 2021; Ham et al. 2021; Hazen et al. 2013; Nichol et al. 2017; Peters et al. 2022). In situations where limited knowledge exists about the ecology of a species, models are a valuable tool for investigating empirical relationships between cetacean distributions and the physical and biological factors (Redfern et al. 2006) and human‐induced threats. For example, SDMs have been used to address the risk of collision (Grossi et al. 2021; Ham et al. 2021; Nichol et al. 2017), overlap with fishing activities (Breen et al. 2016, 2017), changes in distribution (Arcangeli et al. 2013, 2023; Johannessen et al. 2022) and potential climate change impacts (Hazen et al. 2013; Peters et al. 2022). Different modeling techniques are used to study the distribution of cetaceans (Pasanisi et al. 2024), including Boosted Regression Tree (BRT) (Elith et al. 2006; Hazen et al. 2019), Generalized Linear Model (GLM) (McCullagh and Nelder 1989) and Generalized Additive Models (GAM) (Hastie and Tibshirani 1986). The use of GAMS in modeling cetacean distribution allows for non‐linearities in species‐habitat relationships, while the GLM assumes only a linear relationship between the predictors and the response variable. BRTs, instead, are non‐parametric combining regression trees (binary response) and boosting (combining many models to give improved predictive performance) (Elith et al. 2008). With zero‐inflated datasets, as cetacean datasets often are, hurdle models can model presence/absence separate from density. The first component (presence/absence) can be modeled using the binomial model, while the second component (count) is modeled using a zero‐truncated model. By fitting both models individually, the parameters of the hurdle model can be estimated (Asghar et al. 2023; McDowell 2003). This technique has been increasingly employed in recent years to describe the distribution of cetaceans. Moreover, a growing number of studies have demonstrated the superior efficacy of this method (Franchini et al. 2020; Goetz et al. 2012; Jackson‐Ricketts et al. 2020; Johannessen et al. 2022). The growth of techniques for predicting the spatial distribution and abundance of animals has led to a number of comparative studies, often with a focus on assessing prediction accuracy among methods (Elith et al. 2006; Oppel et al. 2012). However, only a limited number of studies have undertaken comparisons specifically for cetacean SDMs (Becker et al. 2020; Sigourney et al. 2020). Such comparative analyses can offer valuable insights into the spatial management of human activities (Becker et al. 2020; Robinson et al. 2011; Thuiller 2004) and enhance our comprehension of both the strengths and limitations of diverse modeling techniques (Brodie et al. 2022; Hazen et al. 2021; Pasanisi et al. 2024), providing guidance for future modeling efforts.

Eight species of cetaceans regularly occur in the Western Mediterranean Sea, especially during summer (Notarbartolo di Sciara 2016). This area is an important feeding ground for marine mammals, given its high productivity from a strong spring bloom (D'Ortenzio and Ribera d'Alcalà 2008). For this reason, in 2002, the Pelagos agreement between Monaco, France, and Italy led to the creation of a Sanctuary for marine mammals. This area also overlaps with human activities such as commercial and passenger ferries, which intensify their activity from June to September (Coomber et al. 2016; David et al. 2011), coinciding with the highest abundance of the animals (Pennino et al. 2017). Because of this vulnerability, in 2024, a Particularly Sensitive Sea Area (PSSA) was created in the North‐western Mediterranean Sea (NWMS) (Resolution MEPC.380(80) 2023), including the entire Pelagos Sanctuary and the Spanish Cetacean Corridor. The second most sighted cetacean is the Mediterranean fin whale ( *Balaenoptera physalus* , Linnaeus, 1758), which is considered globally “Vulnerable” by the IUCN (Cooke 2018), while the subpopulation in the Mediterranean is “Endangered” (Panigada et al. 2021). It is also the only mysticete regularly present in the basin and the most prone to collision with boats (Panigada et al. 2006; Winkler et al. 2020). Hence, identifying reliable SDM tools to describe the spatial and temporal distribution of this species is crucial to inform policy makers and support identifying relevant and effective management measures.

The objective of this study was to compare the predictive power of two commonly used modeling frameworks, GAMs and BRTs, and a two‐stage Hurdle model. Also, we used both presence/absence and the number of individuals as response variables to evaluate how the different parameters explain habitat suitability and the density of the species. Fin whale data were used to develop SDM with the three main goals: (1) define the environmental parameters that influence habitat and density, (2) compare the predictive power of two commonly used and a relatively new modeling framework, and (3) predict the summer distribution in the NWMS.

## 2 Methods and Data

### 2.1 Survey Data

**FIGURE 1:**
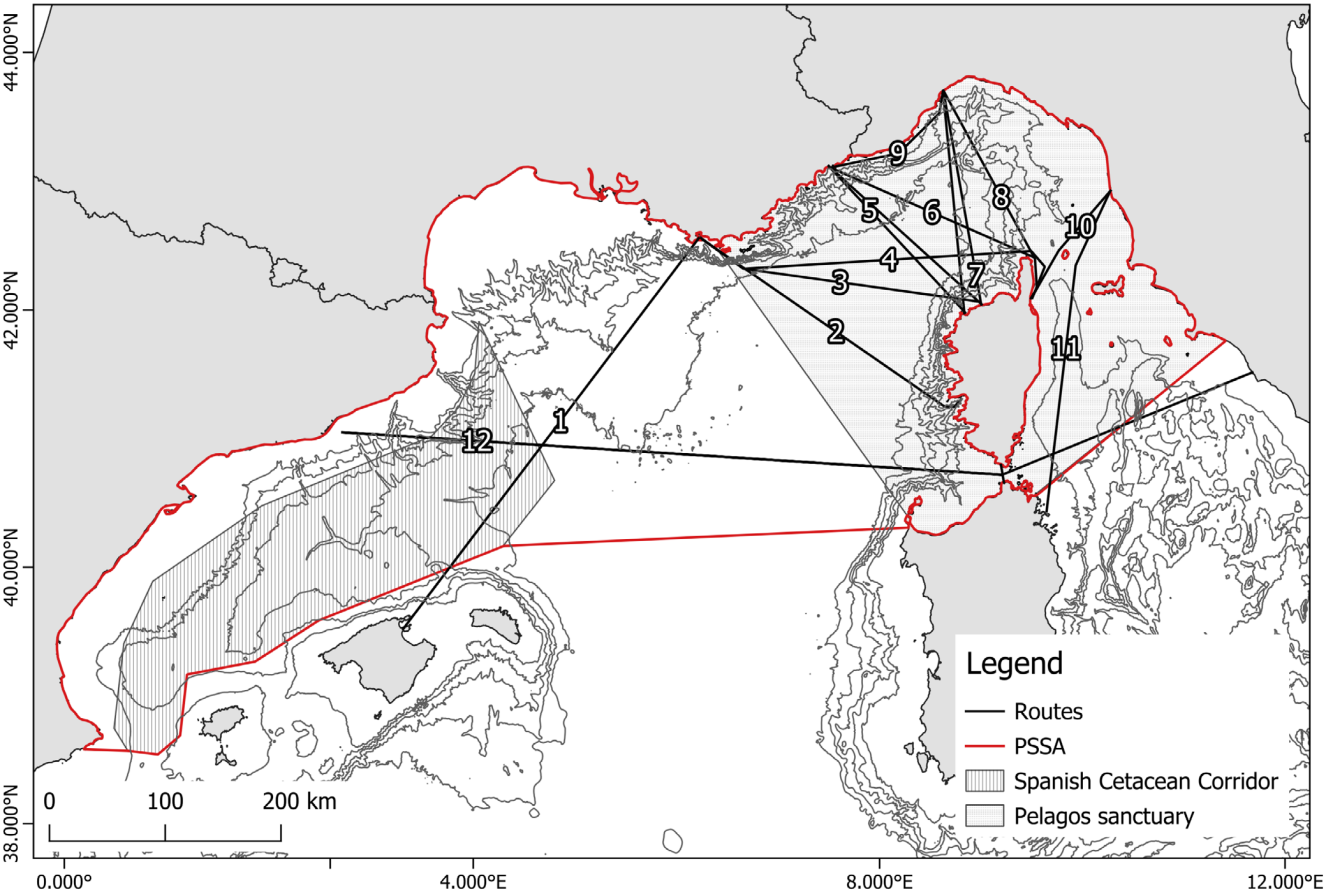
Map of the study area and routes considered, and name of the institution that performed the monitoring. EcoOcéan Institut: (1) Toulon—Alcudia, (2) Toulon —Ajaccio, (3) Toulon—Ile Rousse, (4) Toulon—Bastia, Cima Research Foundation: (5) Nice—Calvi/Ile Rousse, (6) Nice—Bastia, (7) Savona —Calvi/Ile Rousse, (8) Savona—Bastia, (9) Savona—Nice, Univ. Pisa/Accademia del Leviatano/ISPRA: (10) Livorno—Bastia, Univ. Pisa/ISPRA: (11) Livorno—Golfo Aranci, ISPRA/Accademia del Leviatano: (12) Civitavecchia—Barcellona.

The data were obtained from the long‐term monitoring program of the FLT Med Net project. We used fin whale data collected during summer (from the 21st of May to the 30th of September) from 2008 to 2022 on board ferries crossing the north‐western part of the Mediterranean Sea (Figure 1). The data collection in some routes was surveyed also during winter and in the Southern and Eastern parts of the basin, but considering the seasonal migration of the fin whale and the few sightings in these areas, it was decided not to include them in the analysis to avoid possible bias.

Surveys were conducted from the ferries' command deck by a team of at least three MMOs (Marine Mammals Observers) that scanned a sea area of approximately 130° from the bow abeam, to the left and right sides of the command deck by naked eye and using binoculars (7 × 50, fitted with bearing compass and reticle rangefinder) to confirm species and group size. Track line on effort was recorded continuously along the survey using a dedicated Global Positioning System (GPS). Weather conditions were recorded at the beginning of the survey and every time a change occurred. Weather data included wind speed and direction, sea state (following the Beaufort scale), cloud cover, visibility, and rain. The effort was considered only under optimal conditions (Beaufort equal or less than 4, good visibility). Every time a cetacean sighting occurred, the following data were recorded: time, longitude, latitude, radial distance, angle between sighted animal/group and ferry route, species, number of individuals (expressed as minimum, maximum, and best estimation), behavior toward the ferry (indifferent, escaping or approaching) and any peculiar observed behavior. Radial distance was measured using either a rangefinder stick (Wright and Cosentino 2015) or binoculars with a reticle rangefinder. In this latter case, distances were subsequently converted into kilometric distances by applying the formula from Kinzey and Gerrodette (2003) (see Cominelli et al. 2016 for more details on conversion). The angle between the cetacean sighting and ferry route was measured using a compass or a protractor, set with the 0° coinciding with the bow of the ferry.

### 2.2 Data Preparation

GPS points of the ferry track were used to create a transect for each trip, considering a single trip from port to port, after eliminating points where weather conditions were not ideal for fin whale sightings (sea state > 4) (Cominelli et al. 2016) and during which observers were off effort. Data were then overlapped with the 5 km grid created following the European Environmental Agency's INSPIRE compliancy guidelines (EEA 2013). Daily kilometers were calculated for each cell. Sightings collected on effort and in good weather conditions (sea state ≤ 4) were overlapped with the same 5 km grid, and for every cell, the number of individuals was summed daily. If a cell was surveyed less than 1.5 km, and it had no sightings, it was discarded from the analysis.

### 2.3 Data Analysis

#### 2.3.1 Detection Function

The detection function (the probability of animal detection as a function of perpendicular distance) was fitted to the data using the observations from all routes. Different covariates were tested in the model. *Type*: ferries were divided into three categories, as in Tepsich et al. (2020), where Type I included ferries with a height of command deck between 12 and 15 m and an average speed of 17 knots, Type II ferries with 20–22 m command deck heights and an average speed of 23 knots, and Type III with heights between 25 and 29 m and an average speed of 22 knots. *Method*: since the use of binoculars in spotting an animal was hypothesized to have a great impact on the detection of the animals, we also divided the dataset into two categories: survey where protocols require the use of binoculars the whole time, “with binoculars” (B), and where the binoculars are used only for identification, “without binoculars” (A). *Sea state* was also included as a covariate, knowing to affect the distance at which an animal can be spotted. To find the best fitting detection function, multiple candidate models were tested. Key functions include half‐normal (HN), hazard rate (HR), and uniform (U) (Buckland et al. 2015). To reduce the effect of outliers when fitting the detection function, the distance data were truncated at 5% to improve model fit and remove observational outliers (Buckland et al. 2015). Model selection was based on the Akaike Information Criterion (AIC; Sakamoto et al. 1896): different combinations of covariates were tried, and the model with the lowest AIC was retained. Distance sampling analysis has been performed using the package Distance (Miller et al. 2019). The objective of this analysis was the calculation of the ESW (Effective Strip Width) in order to compute the effective Area searched for each cell:

$$A_{i}=2\times L_{i}\times \mathrm{ESW}\times g\left(0\right)$$

where *i* is the cell considered, *L* is the kilometers of effort monitored inside the cell *i* and *g*(0) is the probability of detection of fin whale on the transect line. Based on precedent literature (Palka et al. 2021) and the sightability of the species, *g*(0) was estimated to be 100%.

#### 2.3.2 Habitat Variables

Covariates used in this work can be divided into two categories (Table 1).

**TABLE 1:** Environmental variables used in the models.

	Variables	Code	Unit	Spatial resolution
Static	Bathymetry	Bath	M	30 arc‐seconds (~1 km)
	Slope	Slope	Degrees	30 arc‐seconds (~1 km)
Dynamic	Sea surface temperature	SST	°C	1 km
	Mixed layer depth	MLD	M	1 km
	Chlorophyll (present—lag 1—lag 2)	Chl	Mg/m^3^	1 km
	Finite‐Size Lyapunov Exponents	FSLE	Days^−1^	1 km
	Eddies kinetic energy	EKE	m^2^/s^2^	1 km

Bathymetry is the most common static variable used to describe the distribution of cetaceans (Abrahms et al. 2019; Becker et al. 2020; Cañadas et al. 2023; Grossi et al. 2021), from which we also derived the slope, meaning the angle's degree of the terrain. Bathymetric data have been obtained from the General Bathymetric Chart of the Oceans (GEBCO Compilation Group 2023) which has a resolution of 30 arc‐seconds (~1 km). Bathymetric data were mapped in the 5 km grid, and the mean value was used as the depth of the cell. Regarding the slope, the maximum slope value for each cell was obtained from the 5 km grid.

Dynamic variables including sea surface temperature (SST), Mixed Layer Depth (MLD), and Chlorophyll (Chl) were chosen. Remotely sensed chlorophyll‐a and mixed layer were used as a proxy for productivity, while SST is commonly used to identify prey habitat limitations (Hazen et al. 2017). Chl was considered on the day of the sighting, but also with a lag of 1 (Chl_lag1) and 2 months (Chl_lag2), considering the lag between productivity (spring) and the availability of zooplankton prey (summer) (Littaye et al. 2004). These data were all downloaded from the Copernicus Marine Service System (Volpe et al. 2019; Escudier et al. 2020) and the daily mean was extracted from the 5 km cells.

Also, variables to be used as proxies for mesoscale dynamicity have been considered: eddies kinetic energy (EKE) and Finite‐Size Lyapunov Exponents (FSLE). The Finite‐Size Lyapunov Exponent is a Lagrangian measure of submesoscale circulation downloaded from AVISO (Cotte et al. 2011). Here, we used backward‐in‐time FSLE to identify convergent Lagrangian coherent structures such as fronts, eddies, and upwelling filaments (Scales et al. 2017). We downloaded the *u* and *v* components from Copernicus to calculate EKE (EKE = 0.5*(*U* ^2^ + *V* ^2^), where *U* and *V* are the current components). High EKE is related to the development of eddies and therefore leads to prey aggregation (Arostegui et al. 2022; Woodworth et al. 2012). For these two categories of variables, the daily mean for every 5‐km cell was obtained.

#### 2.3.3 GAM

GAMs were developed in R version 4.3.1 (R Core Team 2023) using package *mgcv* (Wood 2011). Collinearity between variables was tested before being used in the model, and if two variables had more than a 0.6 Pearson correlation value, the one with the lowest AIC was chosen. We used restricted maximum likelihood (REML) to optimize the parameter estimates, and the maximum number of splines was set to 5 to avoid overfitting. The best model was chosen among all the combinations with the lowest Akaike's information criterion (AIC). For the *habitat suitability* model (GAM_HS), we assigned values from 1 to 5 km cells that included sightings and values of 0 to those segments with no sightings. We fit binomial GAMs using a logit link function so that the resultant models describe the probability of species presence, also termed “habitat suitability” For the *density model* (GAM_Dens), we fit a single response model using the number of individuals per cell as the response variable with a tweedie distribution to account for over‐dispersion (Foster and Bravington 2013). The natural log of the effective area searched was included as an offset to account for both varying effort and the different detection probabilities recorded during the surveys.

#### 2.3.4 Hurdle Models

As the third technique, we chose a hurdle model, which models data in two components. The zero component models data as binary with a binomial distribution (zeros vs. all nonzero counts) and the truncated count component models just nonzero counts using a Poisson distribution (Hu et al. 2011). Sightings data were highly zero‐inflated and overdispersed. The hurdle model is an appropriate choice for this study given the nature of our data and its past use in modeling the distribution of marine mammals (Goetz et al. 2012; Gowan and Ortega‐Ortiz 2014; Jackson‐Ricketts et al. 2020). For the binary model, we used the GAM_HS, while for the count model, we used a GAM with a Poisson distribution and using only presence cells and adding as offset the natural logarithm of the effective area surveyed.

#### 2.3.5 BRT

BRTs use machine‐learning methods whereby predictions from single‐tree models are combined to maximize predictive performance (Elith et al. 2008). We fit the BRTs in R (R Core Team 2023) using the package “dismo” version here too, following the methods described in Elith et al. (2008). We built presence–absence BRTs specifying a binomial distribution consistent with the habitat suitability GAMs described above. The BRTs were assigned a tree complexity of 3, a bag fraction of 0.6, and a learning rate (“shrinkage”) of 0.05.

#### 2.3.6 Performance Evaluation and Prediction

Performances were evaluated using different metrics, including *R*‐squared (*R* ^2^), the Area Under the receiver operating characteristic Curve (AUC), and True Skill Statistic (TSS). AUC is a common metric to assess SDM accuracy, with values > 0.75 suggesting the model provides good discrimination between locations where the species is present and where it is absent (Elith et al. 2006). Because of the importance of using multiple metrics for SDM evaluation (Fourcade et al. 2017), predictive performance was evaluated using the AUC and TSS metrics on three training and testing dataset combinations: (a) the full dataset tested, (b) *k*‐fold cross‐validation with a 75%/25% training/testing data split over each of fivefolds, and (c) “Leave One Out” cross‐validation in which a year of data was iteratively left out from training and retained for testing (Abrahms et al. 2019). To calculate AUC and TSS for the GAM density model, we used the sensitivity–specificity sum maximization approach (Liu et al. 2005) to obtain thresholds for species presence. SDM outputs were also compared by visually examining the predicted spatial distributions.

The four models (GAM_HS, GAM_Dens, Hurdle, and BRT) were each used to make predictions on distinct daily composites of environmental conditions for the summer 2008–2022. We used the ensemble of all models to represent expected long‐term patterns in species distributions that account for the varying oceanographic conditions during the 2008–2022 summer cetacean surveys, and the coefficient of variation (CV) to identify the interannual temporal variation of presence/density. The prediction was clipped to the boundaries of the study area to avoid extrapolation.

## 3 Results

In total, 1459 surveys were conducted in summer between 2008 and 2022, providing 217,275 km and 2142 fin whale sightings (2916 individuals) on effort and with sea state ≤ 4. Overlapping with the 5 km grid, 47,071 cells were obtained, where 45,284 were absence cells and 1787 presence cells. The variables considered were not available for every location/time, allowing us to model a total of 46,625 cells, 44,864 absence cells, and 1761 presence cells.

### 3.1 Detection Functions

**FIGURE 2:**
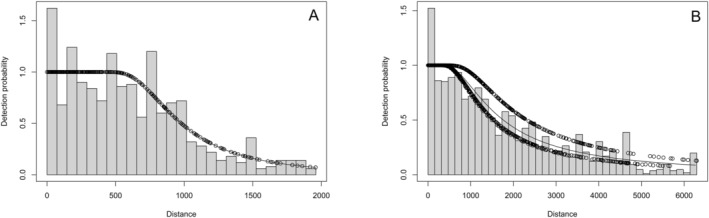
Detection functions for fin whale sightings from (A) monitoring without binoculars and (B) monitoring with binoculars.

The initial AIC‐driven selection highlighted a distinction between the model with the covariate Method (A and B). For this reason, it was decided to divide the dataset in two and to model the detection function separately. The number of observations taken with Method A was 787 and with Method B 1686, both sufficient for modeling. The function with the lowest AIC value for the first subset of the dataset (A) had a hazard rate function and no covariates. Also, for the second subset (B) the function was a hazard rate, and in this case, the covariate of the type of ferry was chosen. From these, we obtained the ESW: 1068 and 2385 m, respectively. Detection function plots are shown in Figure 2.

### 3.2 Modeling

No variables were significantly correlated (Figure S1), allowing all to be included in the model. The results of the best‐fitted models are shown in Table 2. The lowest deviance explained and *R* ^2^ were obtained by the count_model of the hurdle model, with 5% and −0.356 respectively, while the highest deviance explained was obtained by the BRT (29.5%). Depth, SST, MLD, and chl_2lag are the most significant variables in all models, while FSLE was retained only in GAM_HS. The highest deviance explained and *R* ^2^ were obtained by the BRT (29.5%).

**TABLE 2:** Summary of the best‐fitted models.

Model	Predictor variables	Expl. dev.	*R* ^2^
GAM HS (*n* = 46,336)	Bath*** + slope*** + sst*** + fsle. + mld*** + eke* + chl*** + chl_2lag***	11.8%	0.0403
GAM Dens (*n* = 46,336)	Bath*** + slope*** + sst*** + mld*** + chl*** + chl_1lag* + chl_2lag***	14.6%	0.0382
Hurdle_count model (*n* = 1760)	Bath*** + sst + mld + eke* + chl_2lag**	5%	0
BRT (*n* = 46,336)	Bath+slope + sst + fsle+mld + eke+chl + chl_1lag + chl_2lag	29.5%	0.2951

*Note:* BRTs include an estimated *R* ^2^.

Significance codes: ***P < 0.001, **p < 0.01, *p < 0.05.

**FIGURE 3:**
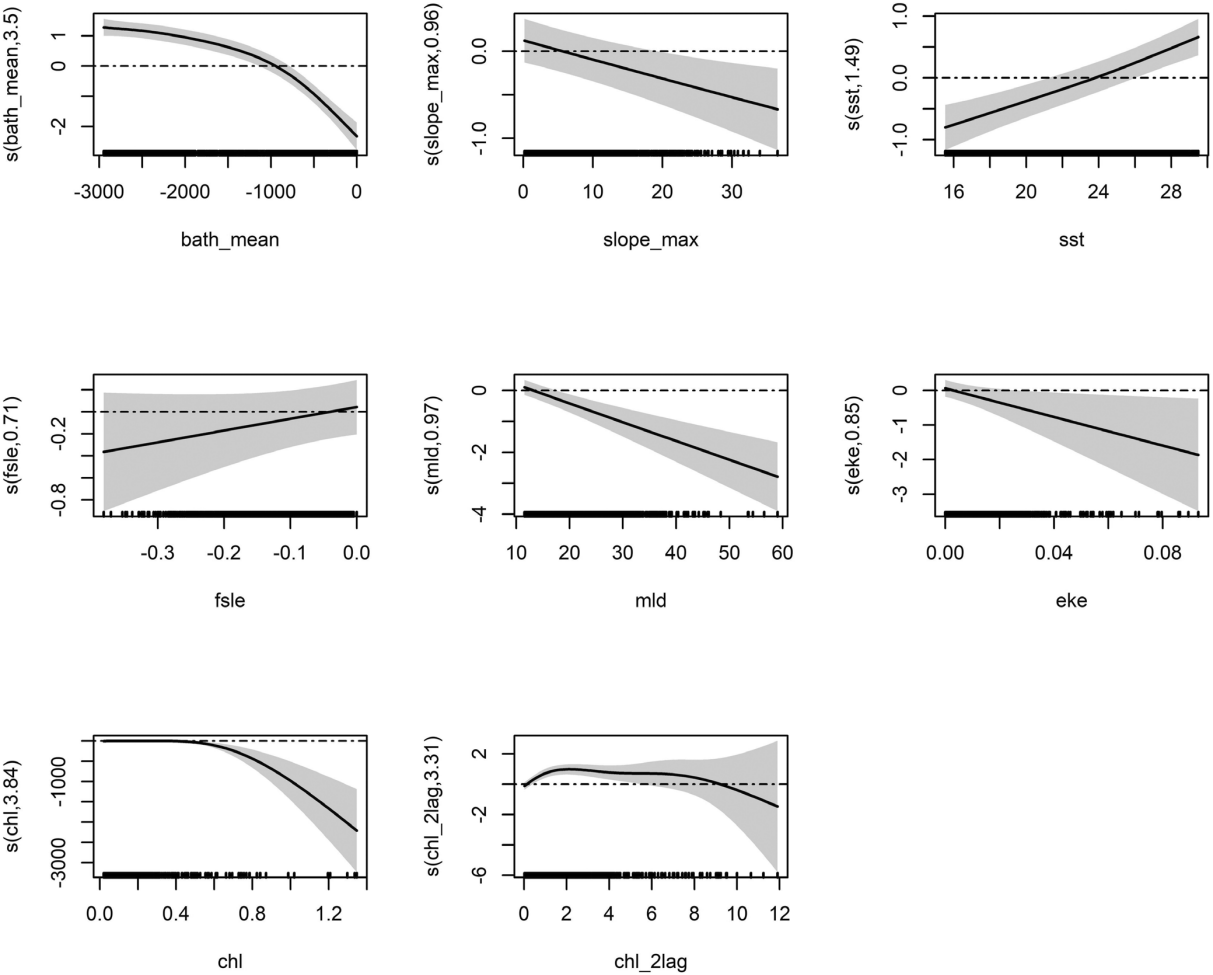
Model function plots of the final GAM of fin whale (GAM_HS). *y* axis represents the fitted function. The *x* axis is in the units of the environmental variable being represented. Shading represents standard errors for the model fit; tick marks on the *x* axis indicate data values within the modeling data.

**FIGURE 4:**
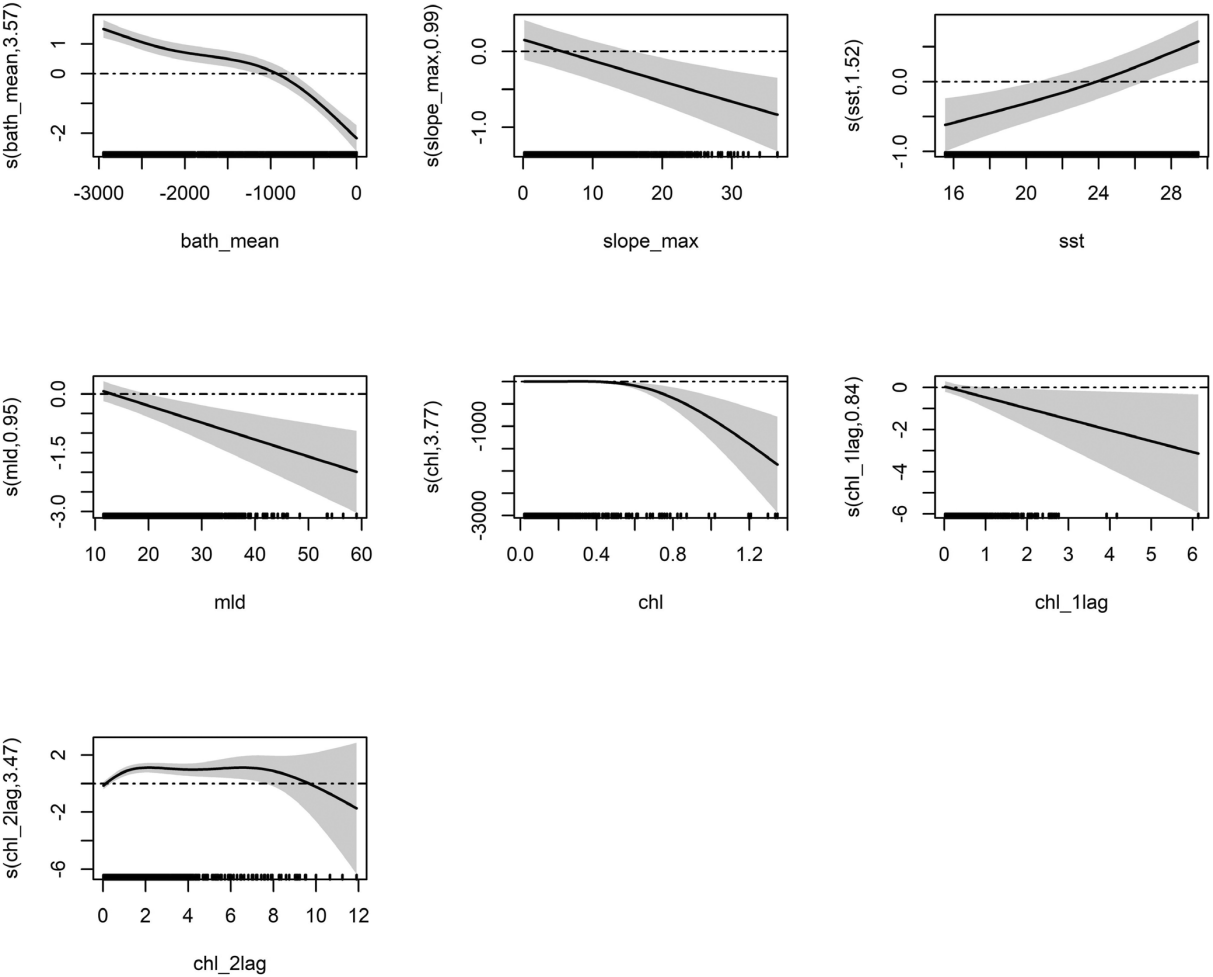
Model function plots of the final GAM of fin whale (GAM_Dens). *y* axis represents the fitted function. The *x* axis is in the units of the environmental variable being represented. Shading represents standard errors for the model fit; tick marks on the *x* axis indicate data values within the modeling data.

Figures 3 and 4 represent GAM‐predicted smooth splines of the occurrence and density of fin whales, respectively, as a function of the explanatory variables. Higher depths were associated with increased presence and density of the fin whale, while the SST showed an opposite trend. The only difference in the chosen variables is in the inclusion of eke and FSLE for GAM_HS, while in GAM_Dens, the chl_1lag was retained. The slope was highly significant and described a preference for flat habitat where the degree is almost zero, meaning that the animals tend to be found and to aggregate where there is no slope. The amount of chlorophyll during summer is close to zero, which could have led to the negative trend, or it could be due to the fact that the animals prefer to hunt in clearer waters. The chlorophyll with a lag of 2 months was highly significant in both models, meaning that in areas with high chlorophyll during winter/spring, the fin whale tends to be present and aggregates months later. Figure 3 Model function plots of the final GAM of fin whale (GAM_HS). The *y* axis represents the fitted function. The *x* axis is in the units of the environmental variable being represented. Shading represents standard errors for the model fit; tick marks on the *x* axis indicate data values within the modeling data whale tend to be present and to aggregate months later.

**FIGURE 5:**
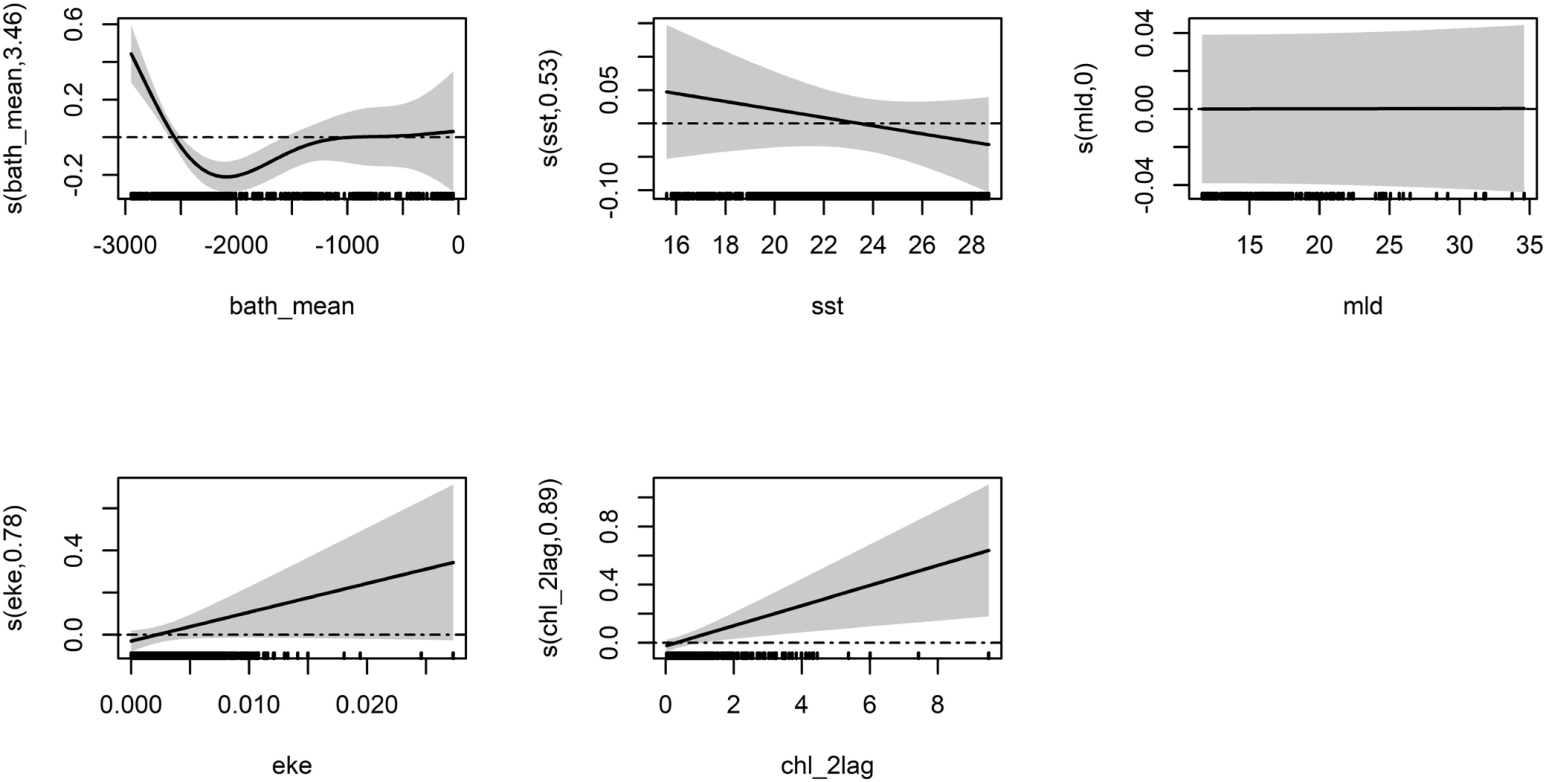
Model function plots of the final GAM of fin whale (Hurdle_count model). *y* axis represents the fitted function. The *x* axis is in the units of the environmental variable being represented. Shading represents standard errors for the model fit; tick marks on the *x* axis indicate data values within the modeling data.

The count_model of the hurdle model (Figure 5), considering only the presence cell of the fin whale, showed a preference for lower SST and higher and lower depth. It means that if the animals are present, they prefer these habitat conditions. The chlorophyll of the lag of 2 months showed the same trend as before, with a positive relation to a higher number of individuals. Here the eke seems to indicate a preference for a higher eke (more dynamicity), even if the variable is not significant.

**FIGURE 6:**
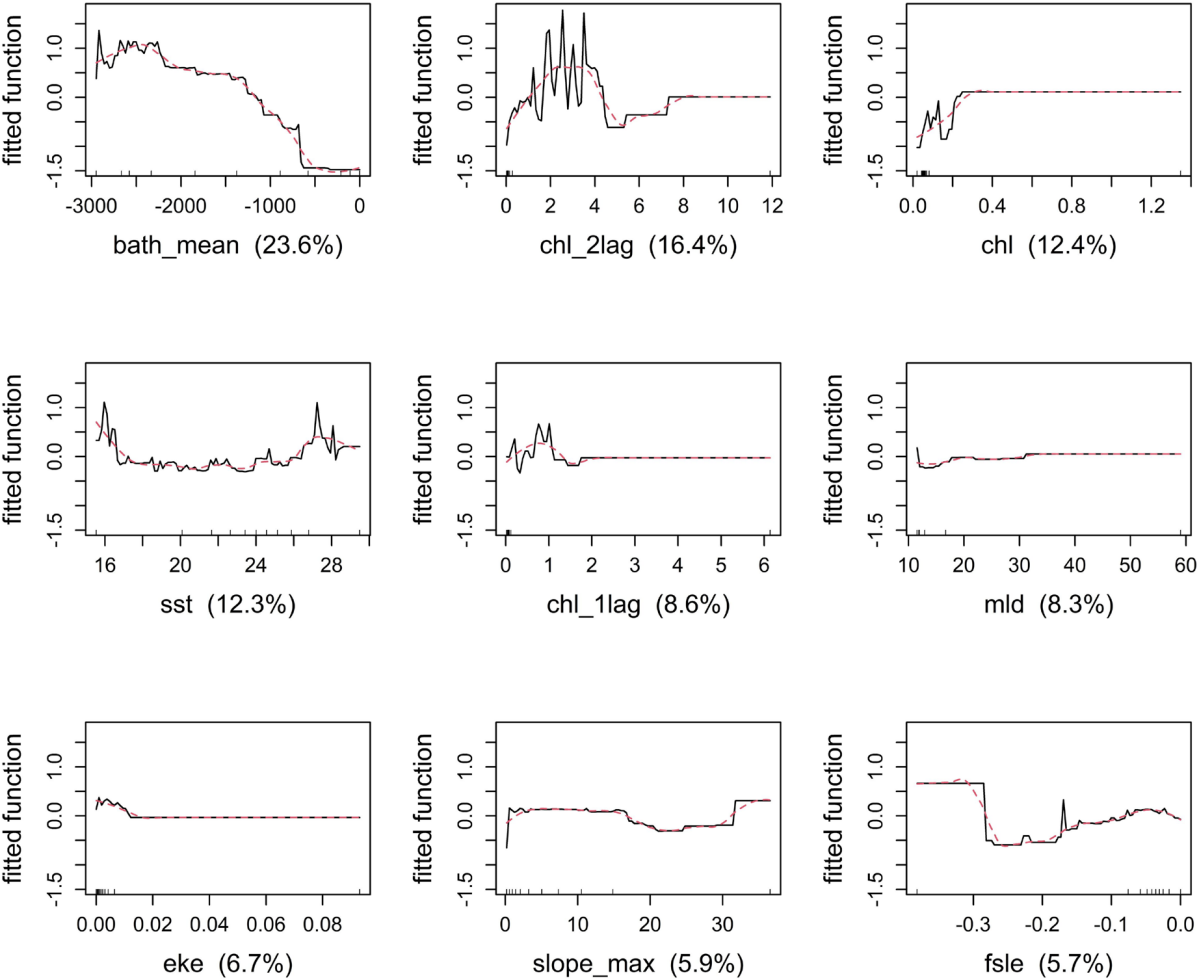
Partial plots for the six most influential variables in the model. *y* axes are on the logit scale and are centered to have zero mean over the data distribution. Rug plots inside below the plots show the distribution of sites across that variable, in deciles.

Looking at the BRT results (Figure 6), the six most influential variables are shown. As for the previous model, bathymetry and chl_2llag are the most important covariates.

Looking at the predictive skill using different training and test datasets (Table 3), all the models showed high performances (AUC > 0.7) The difference between models is minimal. AUC is between 0.76 and 0.77 if training and testing on 100% of the dataset or if dividing it into 25% and 75%; however, the AUC is slightly lower when testing on a single year. As seen from Table S1, there are only 2 years where the AUC is less than 0.6 (2008 and 2020). These are the years with the least survey effort in the entire dataset (beginning of the projects and COVID time), which would probably be the reason for the low predictive power.

**TABLE 3:** Model performance metrics. Area under the receiver operating characteristic curve (AUC) scaled 0–1; True Skill Statistic (TSS) scaled 0–1. The highest‐scoring model for each performance metric is highlighted in bold.

Model	100%		7525 5k		Leave 1 year out	
	AUC	TSS	AUC	TSS	AUC	TSS
GAM HS	0.7783	0.4149	0.7746	0.4258	**0.7127**	**0.3903**
GAM Dens	0.7736	0.4109	0.7672	0.4130	0.7032	0.3847
Hurdle model	0.7782	0.4152	0.7750	0.4229	0.7090	0.3831
BRT	**0.8993**	**0.6275**	**0.9166**	**0.6751**	0.6996	0.3761

### 3.3 Predictive Power

**FIGURE 7:**
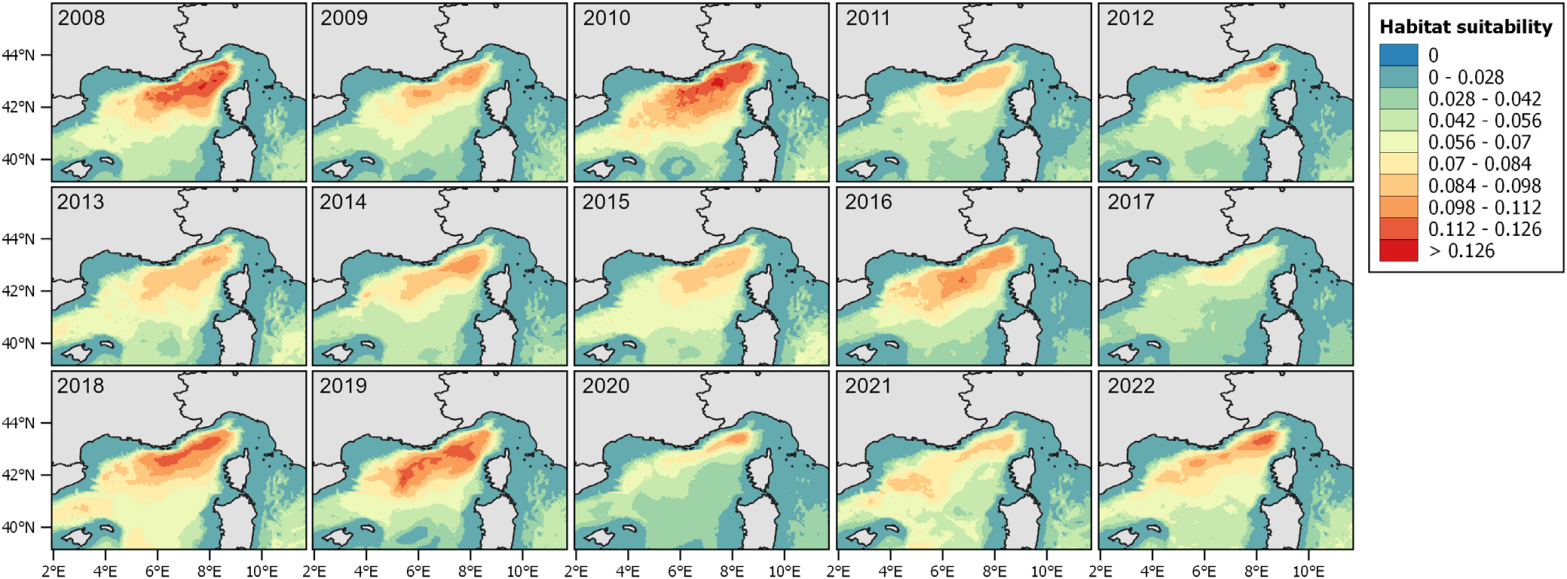
Predicted habitat suitability (GAM_HS) for each year from 2008 to 2022.

Figure 7 shows the summer habitat suitability maps for the study area considered, from 2008 to 2022. The years with higher values are 2008, 2009, 2010, and 2019, while the lowest are 2017 and 2020. In some areas, higher suitability is recurrent, while further south, it varies depending on the year.

**FIGURE 8:**
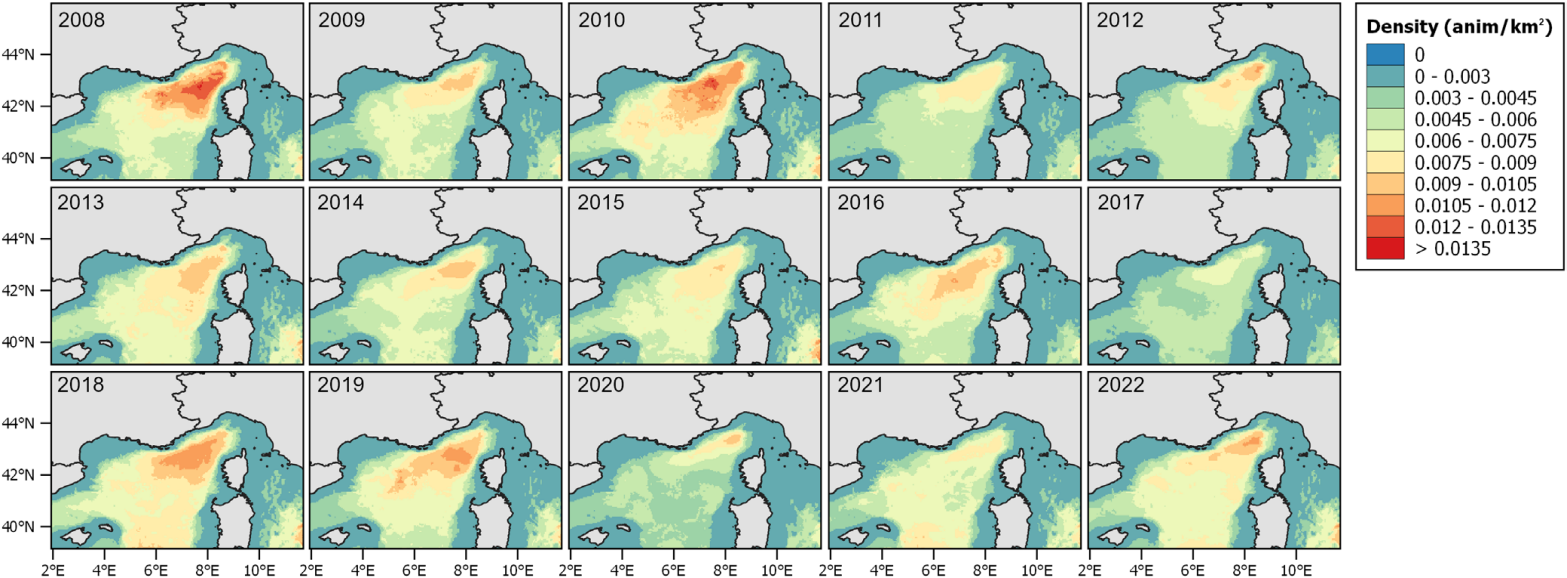
Predicted density values (GAM_Dens) for each year from 2008 to 2022.

Looking at the density model (Figure 8), it reflects the distribution of the fin whale, indicating aggregation in areas where sightings were more common. The areas with higher density are the deepest portion of the Pelagos. The years 2008 and 2010 show the highest values compared to the others, while 2017 and 2020 were the lowest. However, 2017 seems to have the lowest values of density uniformly, while 2020 has some higher values in the north‐western part, closer to the coast.

**FIGURE 9:**
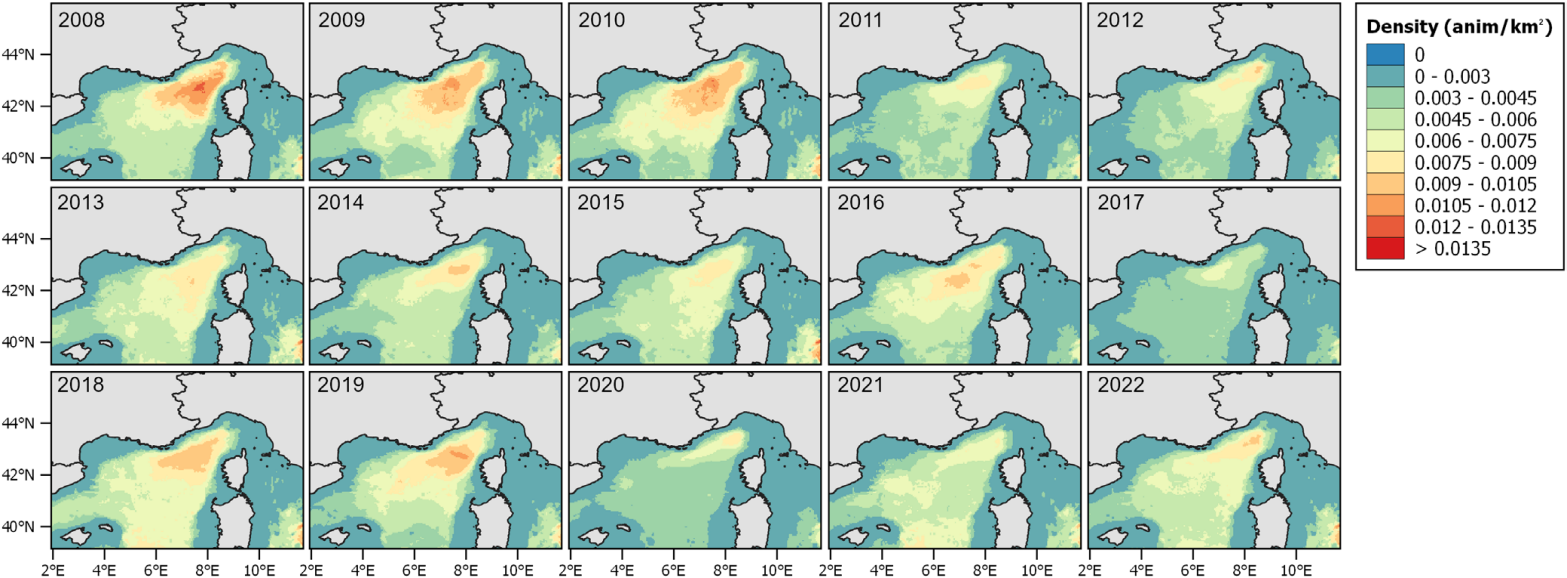
Predicted density obtained with the Hurdle model for each year from 2008 to 2022.

The hurdle model prediction maps (Figure 9) are similar to the density ones, with lower values in some years. The year 2017 is predicted to have fewer animals per kilometer, as well as 2020. The year 2009 here has higher values than the density model, particularly in the north‐central part of the Pelagos.

**FIGURE 10:**
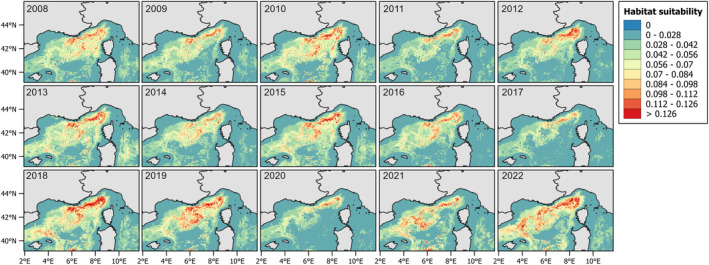
Predicted habitat suitability obtained from BRT for each year from 2008 to 2022.

Regarding the BRTs predictions (Figure 10), the habitat suitability is more scattered than in Figure 5. The interannual variability is less evident, but the years 2020 and 2017 are again the least suitable for the fin whale. The year 2018 is here the year with the highest values. The north‐western area is still the part with the highest habitat suitability throughout the years.

**FIGURE 11:**
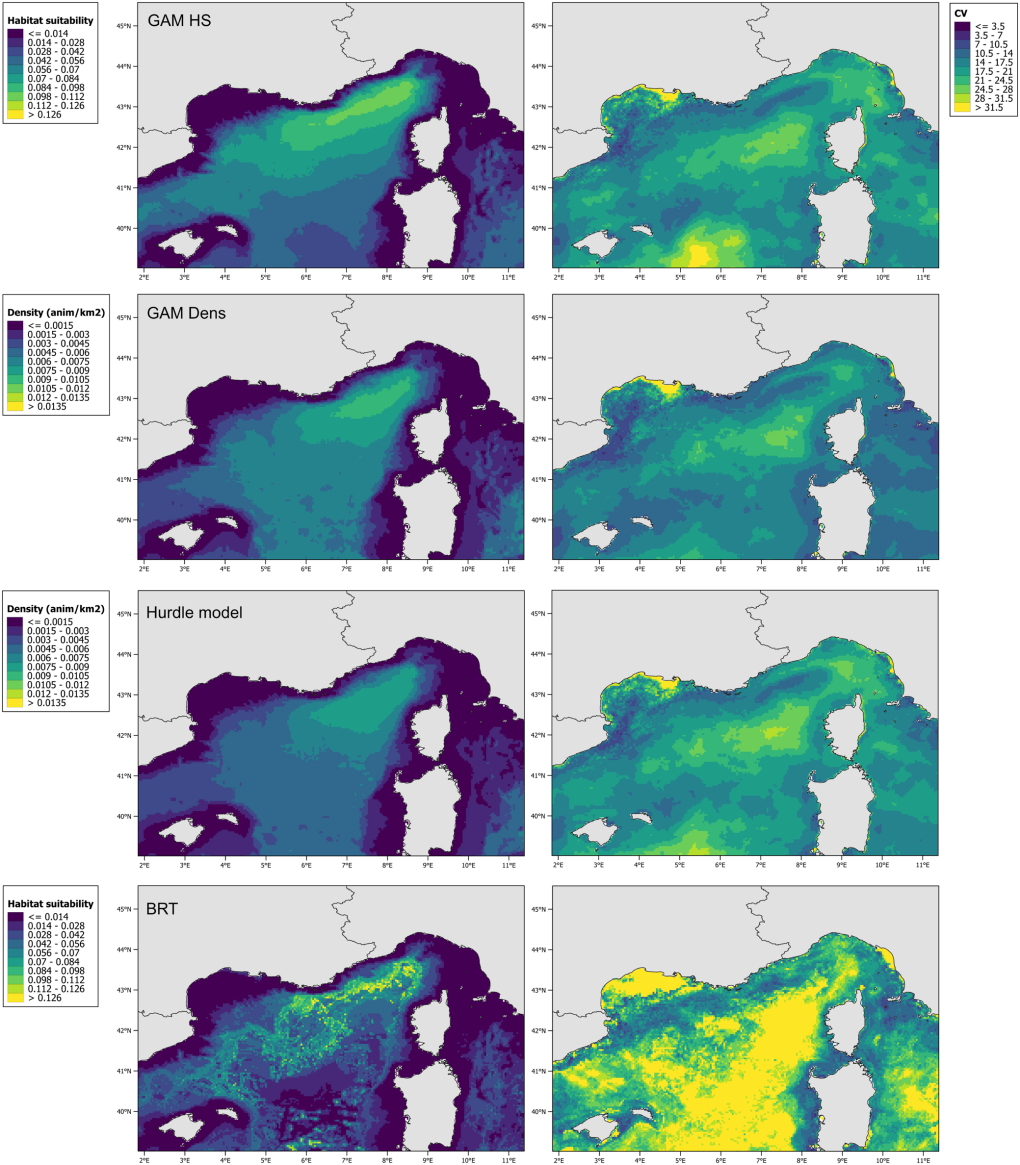
Multi‐year prediction maps and coefficient of variation (CV).

We then ensembled all daily predictions using equal weighting to obtain a multi‐year distribution and CV map for each model (Figure 11). Higher CV indicates greater interannual variability. The habitat suitability map showed a favorable area in the north‐central part of the study area, where most of the sightings were found. However, the CV map had high values in the area just next to Corsica, indicating increased interannual variability in the area. The same hotspot is also predicted in the density and hurdle maps.

## 4 Discussion

### 4.1 Bathymetry and Chlorophyll as Main Driver

In this study, we compared model performance across a variety of modeling techniques, identified strong fin whale –habitat relationships in the north‐western Mediterranean Sea, and created maps of important habitat for use in conservation and management.

In all four models, four variables were consistently retained or considered important: bathymetry, SST, MLD, and chl_2lag. In both GAMs, almost all covariates were significant (except for FSLE in GAM_HS). FSLE was retained only in the GAM_HS but was not significant, while eke was not retained in the density model. The Hurdle_count model retained very few variables, highlighting the importance of the bathymetry and of the chlorophyll present in the area 2 months in advance. The explanatory variables in habitat suitability and density models showed a preference for the pelagic areas, with deep waters (> 1000 m) and a flat sea bottom (slope close to 0). There is a preference for higher sea temperatures (> 24°C) but considering the area and the months used, between May and September, it also coincides with the warmest waters fin the north‐western Mediterranean, where data were collected. Values close to 0 were preferred for the mixed layer depth (both models) and the eddy kinetic energy (Gam_Dens), indicating a preference for less dynamic waters, at least at the surface. Water column stratification might help zooplankton aggregation at depths, indicating a favorable feeding area for the fin whale. The negative trend of the Chl could be explained by the fact that surface chlorophyll is not detected during summer, having already been consumed by the food web; as a consequence, it could also result from increased seawater clarity, which facilitates the predation. On the contrary, sea surface chlorophyll concentration detected 2 months before fin whale presence serves as a valuable proxy of prey availability. We understand that using the chlorophyll levels 1 or 2 months prior to the sightings serves as an indirect indicator of prey availability. However, it performed well in the models and could be a simple and accessible parameter to help explain cetacean distribution. Moreover, being available in advance could be effectively used to forecast species distribution areas during the summer season. Two‐month‐lagged chlorophyll concentration resulted as one of the most significant variables and with a preference for values between 0 and 10 mg/m^3^. Results obtained by all models align with the known habitat preferences for the species in the area. Specifically, bathymetric features were known to be the best describer of the distribution (Aïssi et al. 2015; Morgado et al. 2017; Moulins et al. 2008; Panigada et al. 2008), while other indicators, such as FSLE or MLD, are less informative. Dynamic variables remain important to explain the interannual variability of the species and, in the future, may help predict climate change effects (Grossi et al. 2024).

The hurdle model (count component) retained five variables, where only bathymetry, eke, and chl_2lag were significant. The ideal bathymetry for the density was either shallower than 1000 m or deeper than 2500 m. SST had an opposite trend to the previous GAMS, with a preference for low temperature (< 22°C). Similarly, there was a preference for higher eke values, meaning more energy and dynamicity. This result from the only‐presence model aligns with other studies (Løviknes et al. 2021) that identify colder temperatures as preferred by fin whales. It highlights that SST may be more of a driver for aggregation than presence. While fin whales are more likely to occur in areas with higher SST (since summer is their feeding season), they tend to aggregate and feed in cooler SST waters. Bathymetry is known to be a major driver of this whale's presence, particularly in deep waters. However, our findings reveal that strong aggregation might also occur in shallower areas, pointing out the need for better‐defined conservation strategies considering the highest anthropic impact on such areas.

### 4.2 Predictive Powers and Distribution Maps

The main objective of this work was to compare and understand the predictive power of different Species Distribution Models (SDMs) across multiple response variables. As shown in Table 3, all techniques performed well as evidenced by high AUCs, even when using different testing approaches. Looking at Table S1, the years with the lowest AUC were 2008 and 2020, where the effort was at its minimum. In 2008, the project had just started, and not all the routes were active. In 2020, due to the coronavirus pandemic, the monitoring experienced delays, and it was challenging to conduct the activities. However, if we consider the whole dataset for training and testing, the AUC values are all higher than 0.70, which is consistent with results obtained when we split randomly the dataset in 75%/25%. GAM HS and Hurdle model performed slightly better than the others, but overall, all models demonstrated good predictive skill (Table 3).

Looking at the predictive maps, it becomes evident that there has been a decrease in both presence and density over the years. The interannual variability was more evident in terms of habitat preference, whereas the density values had a clearer decline. The most favorable habitat, characterized by both a high probability of occurrence and extensive spatial coverage, is observed during the early period (2008, 2010) and again in 2018, 2019 (Figure 7, Tables S2 and S3). In contrast, fin whales were predicted to have less suitable habitat in 2017 and 2020. Additionally, during these 2 years, particularly in 2020, it shifted from the western central part of the area, as seen in other years, to a more northern direction. Density predictions mirrored partially the same distribution of the habitat suitability, with the lowest densities observed in 2017 and 2020 again, and the highest density recorded in 2008 and 2010 (Table S3). The prediction obtained from the hurdle model was between the two preceding approaches. While the values are generally lower than those predicted by GAM Dens, the 2017 and 2020 still had the lowest percentage of area (Table S2). BRTs using a different algorithm also resulted in a different predicted distribution (Figure 10), though the overall patterns were consistent with the previous predictions. However, the interannual variability was less evident, and the years with the highest predicted values differed slightly from the GAMs—for example, 2018 had the highest probability values (Table S3).

The density and hurdles predictions appeared to be inadequate in the south‐eastern part of the study area, especially in certain years. The area was once considered to be used just as a north–south migration corridor during the early ‘90th (Marini et al. 1996), but was later recognized as an opportunistic summer feeding ground (Arcangeli et al. 2013; Marini et al. 1996). This area has also been identified as suitable fin whale habitat in multiple models, including BRTs (Druon et al. 2012; Grossi et al. 2021; Ham et al. 2021; Vaes and Druon 2013). The dynamic behavior of the Mediterranean fin whale (Geijer et al. 2016) might limit habitat suitability to only a few months. Nevertheless, it suggests that extrapolation to wider areas must be approached with caution, and seasonal models are more appropriate to capture the dynamics of this migratory and highly mobile species. Using a platform of opportunities offers several advantages: they are cost‐effective, they provide consistent monitoring over long periods, and present an opportunity for public awareness (Evans and Hammond 2004). Even if the routes are determined by touristic demands, which might limit the studied area, all routes crossed all types of habitat, being representative of the area, and low sampling was not considered (Section 2.2). Nonetheless, since the main objective was the comparison of model performances of a variety of modeling techniques, the different survey coverage is not strictly relevant.

Both threshold‐independent (AUC) and threshold‐dependent (TSS) metrics can be misleading when the proportion of the study area occupied by the species is low (Fourcade et al. 2017; Somodi et al. 2017). Our findings align with previous research indicating that AUC alone may not be a reliable indicator of species distribution model (SDM) predictive performance, as it does not account for the spatial distribution of model errors (Lobo et al. 2008).

Looking at the multi‐year averages and CVs (Figure 11), we observe that the deepest part of the north‐western Mediterranean Sea has exhibited the most significant changes over the years. The average from the hurdle model predictions shows low values and less widespread habitat than the density model. The multi‐year average reveals that the different maps tend to overlap, indicating a consistent preference for both presence and density in the north‐western part of the NW Mediterranean Sea.

### 4.3 Density or Habitat Suitability for Management Purpose

The choice of the modeling approach and response variable selection is typically driven by the study's objectives (Braun et al. 2023). Prior to modeling, it is essential to clearly define what is intended to be modeled (the target) and the chosen methodology. In this study, we aimed to examine variability among multiple modeling techniques, seeking common patterns and the most effective approaches for estimating density. Since density GAMs predict abundance, they are well suited to detect changes in animal numbers, especially for species whose distributions contract even when abundance increases, such as dolphins (Becker et al. 2020; Panigada et al. 2017). However, for fin whales, we found that areas of high habitat suitability tend to align with areas of high density. Additionally, the observed interannual variability across all distribution maps (Figures 7, 8, 9, 10) complicates the effort to identify regions of major vulnerability for the species. These maps provide valuable insight about the decline observed over the years, suggesting a potential decrease in the population or a shift toward a more dispersed distribution. From an area‐based management perspective, to inform conservation strategies, maps in Figure 11 are better suited for identifying specific areas: focusing on the core areas could help define new priority areas for conservation. That said, predictions by alternative models can be so variable that they could compromise their use for guiding policy or decision making. A solution to this issue is to use “ensemble” models (Araújo and New 2007). For example, Rowden et al. (2017) combined different habitat suitability models to inform spatial management planning for protecting vulnerable marine ecosystems around New Zealand. Alternatively, as demonstrated by Mi et al. (2017), combining occurrence and abundance data to produce a protection index could help guide the protection of areas with both high occurrence and high abundance.

## 5 Conclusion

Our results provide an improved understanding of the strengths and limitations of GAMs, hurdle models, and BRTs (Table 4). While recognizing the varied strengths and weaknesses of different model types in characterizing species‐environment relationships, our findings underscore the importance of exploring multiple algorithms to better understand species distribution. Furthermore, this study is relevant for dynamic species usually found in small groups (1–2 individuals). We cannot assume that the same results would apply to species like dolphins, which are usually found in large groups or have a wider distribution. Employing a variety of models and response variables could also enhance the ability to identify core areas, which is crucial for effective conservation planning.

**TABLE 4:** Summary of the characteristics of the four models.

Model	Input	Key features	Computation	Accuracy	Strengths	Weaknesses
GAM_HS	Pres/abs	Smooth nonlinear relationships	Uses penalized smooth functions *f*(*X*) in place of linear terms	High	– Easy to interpret – Ideal for a first spatial management plan	– Doesn't take in consideration abundance and aggregation – Model parameters need to be selected carefully
GAM_Dens	Number of individuals			High	– Easy to interpret – Detect changes in animal numbers	– Need for detection function – Might overestimate
BRT	Pres/abs	Ensemble of decision trees	Combines weak learners iteratively, optimizing residuals at each step	High	– No need to test correlation – Easy to perform	– Difficult to interpret (e.g., no *p* values) – Noisy fitted function
Hurdle model	– Pres/abs – Only presence data (number or individuals)	Two‐part model for zero‐inflated data	First component (pres/abs) can be modeled using binomial model, the second component (count) is modeled using a zero‐truncated model (e.g., Poisson)	High	– Ideal for zero‐inflated data – More information about the population (distribution and aggregation)	– Require knowledge of data distribution – More models necessary

## Author Contributions

**Francesca Grossi:** conceptualization (lead), formal analysis (equal), writing – original draft (lead). **Elliott L. Hazen:** conceptualization (supporting), methodology (supporting), supervision (equal), writing – review and editing (equal). **Giulio De Leo:** conceptualization (supporting), formal analysis (supporting), supervision (equal). **Léa David:** data curation (equal), resources (equal), writing – review and editing (equal). **Nathalie Di‐Méglio:** resources (equal). **Antonella Arcangeli:** data curation (equal), resources (equal), writing – review and editing (equal). **Eugenia Pasanisi:** data curation (equal), resources (equal), writing – review and editing (equal). **Ilaria Campana:** resources (equal). **Miriam Paraboschi:** resources (equal). **Alberto Castelli:** resources (equal). **Massimiliano Rosso:** resources (equal), writing – review and editing (equal). **Aurelie Moulins:** data curation (equal), resources (equal). **Paola Tepsich:** resources (equal), supervision (equal), writing – review and editing (equal).

## Conflicts of Interest

The authors declare no conflicts of interest.

## Supporting information


AppendixS1

## Data Availability

The data that support the findings of this study are openly available in Dryad at https://doi.org/10.5061/dryad.d51c5b0ct.

## References

[ece371007-bib-0001] Abrahms, B. , H. Welch , S. Brodie , et al. 2019. “Dynamic Ensemble Models to Predict Distributions and Anthropogenic Risk Exposure for Highly Mobile Species.” Diversity and Distributions 25, no. 8: 1182–1193. 10.1111/ddi.12940.

[ece371007-bib-0002] Aïssi, M. , A. Arcangeli , R. Crosti , et al. 2015. “Cetacean Occurrence and Spatial Distribution in the Central Mediterranean Sea Using Ferries as Platform of Observation.” Russian Journal of Marine Biology 41, no. 5: 343–350. 10.1134/S1063074015050028.

[ece371007-bib-0003] Araújo, M. B. , and M. New . 2007. “Ensemble Forecasting of Species Distributions.” Trends in Ecology & Evolution 22, no. 1: 42–47. 10.1016/J.TREE.2006.09.010. 17011070

[ece371007-bib-0004] Arcangeli, A. , F. Atzori , M. Azzolin , et al. 2023. “Testing Indicators for Trend Assessment of Range and Habitat of Low‐Density Cetacean Species in the Mediterranean Sea.” Frontiers in Marine Science 10: 1116829. 10.3389/fmars.2023.1116829.

[ece371007-bib-0005] Arcangeli, A. , L. Marini , and R. Crosti . 2013. “Changes in Cetacean Presence, Relative Abundance and Distribution Over 20 Years Along a Trans‐Regional Fixed Line Transect in the Central Tyrrhenian Sea.” Marine Ecology 34, no. 1: 112–121. 10.1111/maec.12006.

[ece371007-bib-0006] Arostegui, M. C. , P. Gaube , P. A. Woodworth‐Jefcoats , D. R. Kobayashi , and C. D. Braun . 2022. “Anticyclonic Eddies Aggregate Pelagic Predators in a Subtropical Gyre.” Nature 609, no. 7927: 535–540. 10.1038/s41586-022-05162-6. 36071164

[ece371007-bib-0007] Asghar, M. , S. Ali , and I. Shah . 2023. “Poisson Hurdle Model for Monitoring the Inflation of Zeros.” Quality and Reliability Engineering International 39, no. 6: 2152–2161. 10.1002/QRE.3310.

[ece371007-bib-0008] Becker, E. A. , J. V. Carretta , K. A. Forney , et al. 2020. “Performance Evaluation of Cetacean Species Distribution Models Developed Using Generalized Additive Models and Boosted Regression Trees.” Ecology and Evolution 10, no. 12: 5759–5784. 10.1002/ece3.6316. 32607189 PMC7319248

[ece371007-bib-0009] Braun, C. D. , M. C. Arostegui , N. Farchadi , et al. 2023. “Building Use‐Inspired Species Distribution Models: Using Multiple Data Types to Examine and Improve Model Performance.” Ecological Applications 33, no. 6: 1–20. 10.1002/eap.2893. 37285072

[ece371007-bib-0010] Breen, P. , S. Brown , D. Reid , and E. Rogan . 2016. “Modelling Cetacean Distribution and Mapping Overlap With Fisheries in the Northeast Atlantic.” Ocean and Coastal Management 134: 140–149. 10.1016/j.ocecoaman.2016.09.004.

[ece371007-bib-0011] Breen, P. , S. Brown , D. Reid , and E. Rogan . 2017. “Where Is the Risk? Integrating a Spatial Distribution Model and a Risk Assessment to Identify Areas of Cetacean Interaction With Fisheries in the Northeast Atlantic.” Ocean and Coastal Management 136: 148–155. 10.1016/j.ocecoaman.2016.12.001.

[ece371007-bib-0012] Brodie, S. , J. A. Smith , B. A. Muhling , et al. 2022. “Recommendations for Quantifying and Reducing Uncertainty in Climate Projections of Species Distributions.” Global Change Biology 28, no. 22: 6586–6601. 10.1111/gcb.16371. 35978484 PMC9805044

[ece371007-bib-0013] Buckland, S. T. , E. A. Rexstad , T. A. Marques , and C. S. Oedekoven . 2015. Distance Sampling: Methods and Applications. Springer. 10.1007/978-3-319-19219-2.

[ece371007-bib-0014] Cañadas, A. , N. Pierantonio , J. Gonzalvo , et al. 2023. “Distribution Patterns of Marine Megafauna Density in the Mediterranean Sea Assessed Through the ACCOBAMS Survey Initiative (ASI).” Frontiers in Marine Science 10: 1270917. 10.3389/fmars.2023.1270917.

[ece371007-bib-0015] Cominelli, S. , A. Moulins , M. Rosso , and P. Tepsich . 2016. “Fin Whale Seasonal Trends in the Pelagos Sanctuary, Mediterranean Sea.” Journal of Wildlife Management 80, no. 3: 490–499. 10.1002/jwmg.1027.

[ece371007-bib-0016] Cooke, J. G. 2018. “*Balaenoptera physalus*. The IUCN Red List of Threatened Species 2018: E.T2478A50349982, 8235.” 10.2305/IUCN.UK.2018-2.RLTS.T2478A50349982.en.

[ece371007-bib-0017] Coomber, F. G. , M. D'Incà , M. Rosso , P. Tepsich , and G. Notarbartolo di Sciara . 2016. “Description of the Vessel Traffic Within the North Pelagos Sanctuary: Inputs for Marine Spatial Planning and Management Implications Within an Existing International Marine Protected Area.” Marine Policy 69: 102–113. 10.1016/j.marpol.2016.04.013.

[ece371007-bib-0018] Cotte, C. , F. d'Ovidio , A. Chaigneau , et al. 2011. “Scale‐Dependent Interactions of Mediterranean Whales With Marine Dynamics.” Limnology and Oceanography 56, no. 1: 219–232. 10.4319/lo.2011.56.1.0219.

[ece371007-bib-0019] David, L. , S. Alleaume , and C. Guinet . 2011. “Evaluation of the Potential of Collision Between Fin Whales and Maritime Traffic in the North‐Western Mediterranean Sea in Summer, and Mitigation Solutions.” Journal of Marine Animals and Their Ecology 4, no. 1: 17–28.

[ece371007-bib-0020] D'Ortenzio, F. , and M. Ribera d'Alcalà . 2008. “On the Trophic Regimes of the Mediterranean Sea: A Satellite Analysis.” Biogeosciences Discussions 5, no. 4: 2959–2983. 10.5194/bgd-5-2959-2008.

[ece371007-bib-0021] Druon, J.‐N. , S. Panigada , L. David , et al. 2012. “Potential Feeding Habitat of Fin Whales in the Western Mediterranean Sea: An Environmental Niche Model.” Marine Ecology Progress Series 464: 289–306. 10.3354/meps09810.

[ece371007-bib-0022] Elith, J. , C. H. Graham , R. P. Anderson , et al. 2006. “Novel Methods Improve Prediction of Species' Distributions From Occurrence Data.” Ecography 29, no. 2: 129–151. 10.1111/J.2006.0906-7590.04596.X.

[ece371007-bib-0023] Elith, J. , J. R. Leathwick , and T. Hastie . 2008. “A Working Guide to Boosted Regression Trees.” Journal of Animal Ecology 77, no. 4: 802–813. 10.1111/J.1365-2656.2008.01390.X. 18397250

[ece371007-bib-0024] Escudier, R. , E. Clementi , M. Omar , et al. 2020. “Mediterranean Sea Physical Reanalysis (CMEMS MED‐Currents) (Version 1)” [Data Set]. Copernicus Monitoring Environment Marine Service (CMEMS). 10.25423/CMCC/MEDSEA_MULTIYEAR_PHY_006_004_E3R1.

[ece371007-bib-0025] European Environment Agency . 2013. “EEA Reference Grid for Europe (5km).” https://sdi.eea.europa.eu/catalogue/srv/api/records/c56f5e2b‐6e7f‐4da7‐a5b3‐25a8c17ca717.

[ece371007-bib-0026] Evans, P. G. H. , and P. S. Hammond . 2004. “Monitoring Cetaceans in European Waters.” Mammal Review 34, no. 1: 131–156. 10.1046/j.0305-1838.2003.00027.x.

[ece371007-bib-0027] Foster, S. D. , and M. V. Bravington . 2013. “A Poisson‐Gamma Model for Analysis of Ecological Non‐Negative Continuous Data.” Environmental and Ecological Statistics 20, no. 4: 533–552. 10.1007/S10651-012-0233-0.

[ece371007-bib-0028] Fourcade, Y. , A. G. Besnard , and J. Secondi . 2017. “Paintings Predict the Distribution of Species, or the Challenge of Selecting Environmental Predictors and Evaluation Statistics.” Global Ecology and Biogeography 27, no. 2: 245–256. 10.1111/GEB.12684.

[ece371007-bib-0029] Franchini, F. , S. Smout , C. Blight , et al. 2020. “Habitat Partitioning in Sympatric Delphinids Around the Falkland Islands: Predicting Distributions Based on a Limited Data Set.” Frontiers in Marine Science 7: 277. 10.3389/FMARS.2020.00277.

[ece371007-bib-0030] GEBCO Compilation Group . 2023. “GEBCO 2023 Grid.” 10.5285/f98b053b-0cbc-6c23-e053-6c86abc0af7b.

[ece371007-bib-0031] Geijer, C. K. A. , G. N. di Sciara , and S. Panigada . 2016. “Mysticete Migration Revisited: Are Mediterranean Fin Whales an Anomaly?” Mammal Review 46, no. 4: 284–296. 10.1111/mam.12069.

[ece371007-bib-0032] Goetz, K. T. , R. A. Montgomery , J. M. V. Ver Hoef , R. C. Hobbs , and D. S. Johnson . 2012. “Identifying Essential Summer Habitat of the Endangered Beluga Whale *Delphinapterus leucas* in Cook Inlet, Alaska.” Endangered Species Research 16, no. 2: 135–147. 10.3354/esr00394.

[ece371007-bib-0033] Gowan, T. A. , and J. G. Ortega‐Ortiz . 2014. “Wintering Habitat Model for the North Atlantic Right Whale (*Eubalaena glacialis*) in the Southeastern United States.” PLoS One 9, no. 4: e95126. 10.1371/JOURNAL.PONE.0095126.G002. 24740091 PMC3989274

[ece371007-bib-0034] Grossi, F. , M. Lagasio , A. Napoli , A. Provenzale , and P. Tepsich . 2024. “Phytoplankton Spring Bloom in the NW Mediterranean Sea Under Climate Change.” Science of the Total Environment 914: 169884. 10.1016/j.scitotenv.2024.169884. 38190897

[ece371007-bib-0035] Grossi, F. , E. Lahaye , A. Moulins , A. Borroni , M. Rosso , and P. Tepsich . 2021. “Locating Ship Strike Risk Hotspots for Fin Whale (*Balaenoptera physalus*) and Sperm Whale (*Physeter macrocephalus*) Along Main Shipping Lanes in the North‐Western Mediterranean Sea.” Ocean and Coastal Management 212: 105820. 10.1016/J.OCECOAMAN.2021.105820.

[ece371007-bib-0036] Ham, G. S. , E. Lahaye , M. Rosso , A. Moulins , E. Hines , and P. Tepsich . 2021. “Predicting Summer Fin Whale Distribution in the Pelagos Sanctuary (North‐Western Mediterranean Sea) to Identify Dynamic Whale–Vessel Collision Risk Areas.” Aquatic Conservation: Marine and Freshwater Ecosystems 31, no. 8: 2257–2277. 10.1002/aqc.3614.

[ece371007-bib-0037] Hastie, T. , and R. Tibshirani . 1986. “Generalized Additive Models.” Statistical Science 1, no. 3: 297–310. 10.1214/ss/1177013604. 8548102

[ece371007-bib-0038] Hazen, E. L. , B. Abrahms , S. Brodie , et al. 2019. “Marine Top Predators as Climate and Ecosystem Sentinels.” Frontiers in Ecology and the Environment 17, no. 10: 565–574. 10.1002/fee.2125.

[ece371007-bib-0039] Hazen, E. L. , B. Abrahms , S. Brodie , G. Carroll , H. Welch , and S. J. Bograd . 2021. “Where Did They Not Go? Considerations for Generating Pseudo‐Absences for Telemetry‐Based Habitat Models.” Movement Ecology 9, no. 1: 1–13. 10.1186/s40462-021-00240-2. 33596991 PMC7888118

[ece371007-bib-0040] Hazen, E. L. , S. Jorgensen , R. R. Rykaczewski , et al. 2013. “Predicted Habitat Shifts of Pacific Top Predators in a Changing Climate.” Nature Climate Change 3, no. 3: 234–238. 10.1038/nclimate1686.

[ece371007-bib-0041] Hazen, E. L. , D. M. Palacios , K. A. Forney , et al. 2017. “WhaleWatch: A Dynamic Management Tool for Predicting Blue Whale Density in the California Current.” Journal of Applied Ecology 54, no. 5: 1415–1428. 10.1111/1365-2664.12820.

[ece371007-bib-0042] Hu, M. C. , M. Pavlicova , and E. V. Nunes . 2011. “Zero‐Inflated and Hurdle Models of Count Data With Extra Zeros: Examples From an HIV‐Risk Reduction Intervention Trial.” American Journal of Drug and Alcohol Abuse 37, no. 5: 367–375. 10.3109/00952990.2011.597280. 21854279 PMC3238139

[ece371007-bib-0043] Jackson‐Ricketts, J. , C. Junchompoo , E. M. Hines , et al. 2020. “Habitat Modeling of Irrawaddy Dolphins (*Orcaella brevirostris*) in the Eastern Gulf of Thailand.” Ecology and Evolution 10, no. 6: 2778–2792. 10.1002/ece3.6023. 32211155 PMC7083678

[ece371007-bib-0044] Johannessen, J. E. D. , M. Biuw , U. Lindstrøm , et al. 2022. “Intra‐Season Variations in Distribution and Abundance of Humpback Whales in the West Antarctic Peninsula Using Cruise Vessels as Opportunistic Platforms.” Ecology and Evolution 12, no. 2: 1–13. 10.1002/ece3.8571. PMC8826076 35154653

[ece371007-bib-0045] Kinzey, D. , and T. Gerrodette . 2003. “Distance Measurements Using Binoculars From Ships at Sea: Accuracy, Precision and Effects of Refraction.” Journal of Cetacean Research and Management 5, no. 2: 159–171. 10.47536/jcrm.v5i2.816.

[ece371007-bib-0046] Littaye, A. , A. Gannier , S. Laran , and J. P. F. Wilson . 2004. “The Relationship Between Summer Aggregation of Fin Whales and Satellite‐Derived Environmental Conditions in the Northwestern Mediterranean Sea.” Remote Sensing of Environment 90, no. 1: 44–52. 10.1016/j.rse.2003.11.017.

[ece371007-bib-0047] Liu, C. , P. M. Berry , T. P. Dawson , et al. 2005. “Selecting Thresholds of Occurrence in the Prediction of Species Distributions.” Ecography 28, no. 3: 385–393. 10.1111/J.0906-7590.2005.03957.X.

[ece371007-bib-0048] Lobo, J. M. , A. Jiménez‐valverde , and R. Real . 2008. “AUC: A Misleading Measure of the Performance of Predictive Distribution Models.” Global Ecology and Biogeography 17, no. 2: 145–151. 10.1111/J.1466-8238.2007.00358.X.

[ece371007-bib-0049] Løviknes, S. , K. H. Jensen , B. A. Krafft , V. Anthonypillai , and L. Nøttestad . 2021. “Feeding Hotspots and Distribution of Fin and Humpback Whales in the Norwegian Sea From 2013 to 2018.” Frontiers in Marine Science 8: 6. 10.3389/fmars.2021.632720.

[ece371007-bib-0050] Marini, L. , C. Consiglio , A. M. Angradi , et al. 1996. “Distribution, Abundance and Seasonality of Cetaceans Sighted During Scheduled Ferry Crossings in the Central Tyrrhenian Sea: 1989–1992.” Italian Journal of Zoology 63, no. 4: 381–388. 10.1080/11250009609356163.

[ece371007-bib-0051] McCullagh, P. , and J. A. Nelder . 1989. Generalized Linear Models. 2nd ed. L. Chapman & Hall.

[ece371007-bib-0052] McDowell, A. 2003. “From the Help Desk: Hurdle Models.” Stata Journal: Promoting Communications on Statistics and Stata 3, no. 2: 178–184. 10.1177/1536867x0300300207.

[ece371007-bib-0053] Mi, C. , F. Huettmann , R. Sun , and Y. Guo . 2017. “Combining Occurrence and Abundance Distribution Models for the Conservation of the Great Bustard.” PeerJ 5: e4160. 10.7717/peerj.4160. 29255652 PMC5732545

[ece371007-bib-0054] Miller, D. L. , E. Rexstad , L. Thomas , J. L. Laake , and L. Marshall . 2019. “Distance Sampling in R.” Journal of Statistical Software 89, no. 1: 1–28. 10.18637/JSS.V089.I01.

[ece371007-bib-0055] Morgado, C. , A. Martins , M. Rosso , A. Moulins , and P. Tepsich . 2017. “Fin Whale Presence and Distribution in the Pelagos Sanctuary: Temporal and Spatial Variability Along 2 Fixed‐Line Transects Monitored in 2009‐2013.” International Journal of Marine and Environmental Sciences 1: 1–14.

[ece371007-bib-0056] Moulins, A. , M. Rosso , M. Ballardini , and M. Würtz . 2008. “Partitioning of the Pelagos Sanctuary (North‐Western Mediterranean Sea) Into Hotspots and Coldspots of Cetacean Distributions.” Journal of the Marine Biological Association of the United Kingdom 88, no. 6: 1273–1281. 10.1017/S0025315408000763.

[ece371007-bib-0057] Nichol, L. , B. Wright , P. O'Hara , and J. Ford . 2017. “Risk of Lethal Vessel Strikes to Humpback and Fin Whales off the West Coast of Vancouver Island, Canada.” Endangered Species Research 32: 373–390. 10.3354/esr00813.

[ece371007-bib-0058] Notarbartolo di Sciara, G. 2016. “Chapter One – Marine Mammals in the Mediterranean Sea: An Overview.” In Advances in Marine Biology, edited by G. Notarbartolo Di Sciara , M. Podestà , and B. E. Curry , Vol. 75, 1–36. Academic Press. 10.1016/bs.amb.2016.08.005. 27770981

[ece371007-bib-0059] Oppel, S. , A. Meirinho , I. Ramírez , et al. 2012. “Comparison of Five Modelling Techniques to Predict the Spatial Distribution and Abundance of Seabirds.” Biological Conservation 156: 94–104. 10.1016/j.biocon.2011.11.013.

[ece371007-bib-0060] Palka, D. , L. Aichinger Dias , E. Broughton , et al. 2021. “Atlantic Marine Assessment Program for Protected Species: FY15‐FY19.” May, 4 p. http://www.boem.gov/Atlantic‐Marine‐Asse. http://www.boem.gov/Atlantic‐Marine‐Assessment‐Program‐for‐Protected‐Species‐II/.

[ece371007-bib-0061] Panigada, S. , P. Gauffier , and G. Notarbartolo di Sciara . 2021. “*Balaenoptera physalus* (Mediterranean Subpopulation). The IUCN Red List of Threatened Species 2021: E.T16208224A50387979.” 10.2305/IUCN.UK.2021-3.RLTS.T16208224A50387979.en.

[ece371007-bib-0062] Panigada, S. , G. Lauriano , G. Donovan , et al. 2017. “Estimating Cetacean Density and Abundance in the Central and Western Mediterranean Sea Through Aerial Surveys: Implications for Management.” Deep Sea Research Part II: Topical Studies in Oceanography 141: 41–58. 10.1016/J.DSR2.2017.04.018.

[ece371007-bib-0063] Panigada, S. , G. Pesante , M. Zanardelli , F. Capoulade , A. Gannier , and M. T. Weinrich . 2006. “Mediterranean Fin Whales at Risk From Fatal Ship Strikes.” Marine Pollution Bulletin 52, no. 10: 1287–1298. 10.1016/j.marpolbul.2006.03.014. 16712877

[ece371007-bib-0064] Panigada, S. , M. Zanardelli , M. MacKenzie , C. Donovan , F. Mélin , and P. S. Hammond . 2008. “Modelling Habitat Preferences for Fin Whales and Striped Dolphins in the Pelagos Sanctuary (Western Mediterranean Sea) With Physiographic and Remote Sensing Variables.” Remote Sensing of Environment 112, no. 8: 3400–3412. 10.1016/j.rse.2007.11.017.

[ece371007-bib-0065] Pasanisi, E. , D. S. Pace , A. Orasi , M. Vitale , and A. Arcangeli . 2024. “A Global Systematic Review of Species Distribution Modelling Approaches for Cetaceans and Sea Turtles.” Ecological Informatics 82: 102700. 10.1016/j.ecoinf.2024.102700.

[ece371007-bib-0066] Pennino, M. G. , A. Arcangeli , V. Prado Fonseca , et al. 2017. “A Spatially Explicit Risk Assessment Approach: Cetaceans and Marine Traffic in the Pelagos Sanctuary (Mediterranean Sea).” PLoS One 12, no. 6: e0179686. 10.1371/journal.pone.0179686. 28644882 PMC5482452

[ece371007-bib-0067] Peters, K. J. , K. A. Stockin , and F. Saltré . 2022. “On the Rise: Climate Change in New Zealand Will Cause Sperm and Blue Whales to Seek Higher Latitudes.” Ecological Indicators 142: 109235. 10.1016/j.ecolind.2022.109235.

[ece371007-bib-0068] R Core Team . 2023. R: A Language and Environment for Statistical Computing. R Foundation for Statistical Computing. https://www.R‐project.org/.

[ece371007-bib-0069] Redfern, J. , M. Ferguson , E. Becker , et al. 2006. “Techniques for Cetacean‐Habitat Modeling.” Marine Ecology Progress Series 310: 271–295.

[ece371007-bib-0070] Resolution MEPC.380(80) . Adopted on 7 July 2023. “Designation of the North‐Western Mediterranean Sea as a Particularly Sensitive Sea Area.” 1–24.

[ece371007-bib-0071] Robinson, L. M. , J. Elith , A. J. Hobday , et al. 2011. “Pushing the Limits in Marine Species Distribution Modelling: Lessons From the Land Present Challenges.” Global Ecology and Biogeography 20, no. 6: 789–802. 10.1111/j.1466-8238.2010.00636.x.

[ece371007-bib-0072] Rowden, A. A. , O. F. Anderson , S. E. Georgian , et al. 2017. “High‐Resolution Habitat Suitability Models for the Conservation and Management of Vulnerable Marine Ecosystems on the Louisville Seamount Chain, South Pacific Ocean.” Frontiers in Marine Science 4: 335. 10.3389/fmars.2017.00335.

[ece371007-bib-0073] Sakamoto, Y. , M. Ishiguro , and G. Kitagawa . 1896. Akaike Information Criterion Statistics. Vol. 81. D. Reidel.

[ece371007-bib-0074] Scales, K. L. , G. S. Schorr , E. L. Hazen , et al. 2017. “Should I Stay or Should I Go? Modelling Year‐Round Habitat Suitability and Drivers of Residency for Fin Whales in the California Current.” Diversity and Distributions 23, no. 10: 1204–1215. 10.1111/DDI.12611.

[ece371007-bib-0075] Sigourney, D. B. , S. Chavez‐Rosales , P. B. Conn , L. Garrison , E. Josephson , and D. Palka . 2020. “Developing and Assessing a Density Surface Model in a Bayesian Hierarchical Framework With a Focus on Uncertainty: Insights From Simulations and an Application to Fin Whales (*Balaenoptera physalus*).” PeerJ 8: e8226. 10.7717/peerj.8226. 32002319 PMC6983298

[ece371007-bib-0076] Somodi, I. , N. Lepesi , and Z. Botta‐Dukát . 2017. “Prevalence Dependence in Model Goodness Measures With Special Emphasis on True Skill Statistics.” Ecology and Evolution 7, no. 3: 863–872. 10.1002/ECE3.2654. 28168023 PMC5288248

[ece371007-bib-0077] Tepsich, P. , I. Schettino , F. Atzori , et al. 2020. “Trends in Summer Presence of Fin Whales in the Western Mediterranean Sea Region: New Insights From a Long‐Term Monitoring Program.” PeerJ 8: e10544. 10.7717/peerj.10544. 33362978 PMC7745674

[ece371007-bib-0078] Thuiller, W. 2004. “Patterns and Uncertainties of Species' Range Shifts Under Climate Change.” Global Change Biology 10, no. 12: 2020–2027. 10.1111/J.1365-2486.2004.00859.X. PMC4340562 25200514

[ece371007-bib-0079] Vaes, T. , and J. N. Druon . 2013. “Mapping of Potential Risk of Ship Strike With Fin Whales in the Western Mediterranean Sea: A Scientific and Technical Review Using the Potential Habitat of Fin Whales and the Effective Vessel Density.” JRC Scientific and Policy Reports. https://data.europa.eu/doi/10.2788/8520.

[ece371007-bib-0080] Volpe, G. , S. Colella , V. E. Brando , et al. 2019. “Mediterranean Ocean Colour Level 3 Operational Multi‐Sensor Processing.” Ocean Science 15, no. 1: 127–146.

[ece371007-bib-0081] Winkler, C. , S. Panigada , S. Murphy , and F. Ritter . 2020. “Global Numbers of Ship Strikes: An Assessment of Collisions Between Vessels and Cetaceans Using Available Data in the IWC Ship Strike Database.” IWC B, 68.

[ece371007-bib-0082] Wood, S. N. 2011. “Fast Stable Restricted Maximum Likelihood and Marginal Likelihood Estimation of Semiparametric Generalized Linear Models.” Journal of the Royal Statistical Society: Series B (Statistical Methodology) 73, no. 1: 3–36. 10.1111/j.1467-9868.2010.00749.x.

[ece371007-bib-0083] Woodworth, P. A. , G. S. Schorr , R. W. Baird , et al. 2012. “Eddies as Offshore Foraging Grounds for Melon‐Headed Whales (*Peponocephala electra*).” Marine Mammal Science 28, no. 3: 638–647. 10.1111/j.1748-7692.2011.00509.x.

[ece371007-bib-0084] Wright, A. J. , and A. M. Cosentino . 2015. “JNCC Guidelines for Minimising the Risk of Injury and Disturbance to Marine Mammals From Seismic Surveys: We Can Do Better.” Marine Pollution Bulletin 100, no. 1: 231–239. 10.1016/J.MARPOLBUL.2015.08.045. 26364203

