# Supplementary material for: Evaluating Three Modelling Frameworks for Assessing Changes in Fin Whale Distribution in the Mediterranean Sea
Source: Ecol Evol. 2025 Mar 7;15(3):e71007. doi: 10.1002/ece3.71007 (PMC11886417; doi:10.1002/ece3.71007)
Supplement: Supplementary file 1 — AppendixS1 [file ECE3-15-e71007-s001.docx]

**Supplementary material**

**Figure S1. Correlation plots between variables used in the models**

**
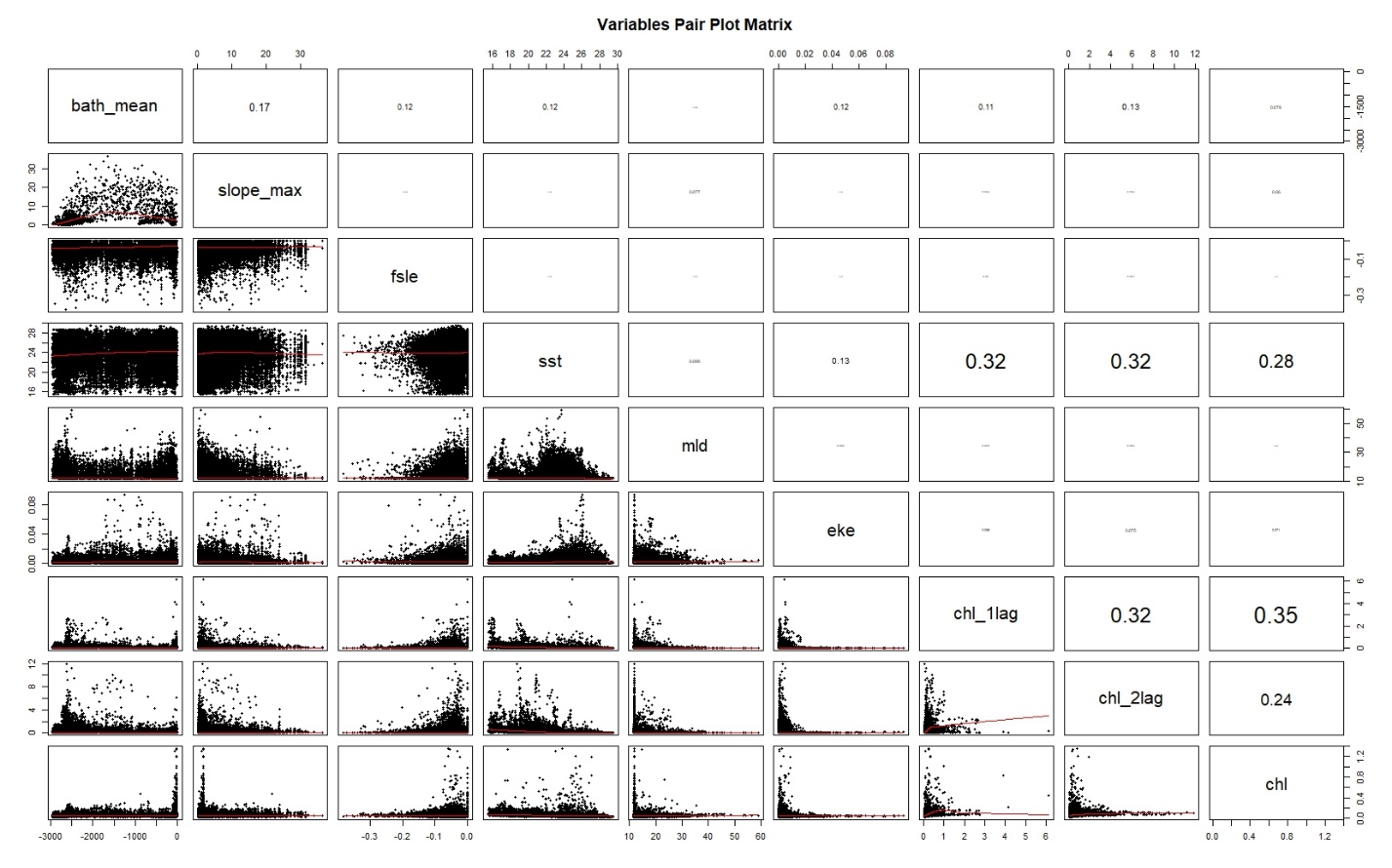
**

**Table S1. Model performance: “Leave One Out” cross‐validation in which a year of data was iteratively left out from training and retained for testing**

| **Year** | **GAM_HS** | | **Gam_dens** | | **Hurdle model** | | **BRT** | |
| --- | --- | --- | --- | --- | --- | --- | --- | --- |
|  | **AUC** | **TSS** | **AUC** | **TSS** | **AUC** | **TSS** | **AUC** | **TSS** |
| *2008* | 0.560748 | 0.4042056 | 0.3306075 | 0.1378505 | 0.4065421 | 0.261682 | 0.297897 | 0.09813 |
| *2009* | 0.765757 | 0.4910631 | 0.8312448 | 0.5934807 | 0.7780171 | 0.492318 | 0.762975 | 0.52585 |
| *2010* | 0.682764 | 0.328697 | 0.6899377 | 0.343177 | 0.6924755 | 0.34168 | 0.69349 | 0.30432 |
| *2011* | 0.790658 | 0.5341055 | 0.7801074 | 0.4887635 | 0.7850788 | 0.493416 | 0.742647 | 0.40689 |
| *2012* | 0.801245 | 0.5417019 | 0.8064338 | 0.503256 | 0.8137495 | 0.517883 | 0.821 | 0.50273 |
| *2013* | 0.767638 | 0.4057658 | 0.7754972 | 0.410843 | 0.776052 | 0.411047 | 0.73569 | 0.39306 |
| *2014* | 0.662318 | 0.2645829 | 0.670961 | 0.2953976 | 0.6722794 | 0.282862 | 0.672681 | 0.25557 |
| *2015* | 0.793685 | 0.4956052 | 0.8057189 | 0.478039 | 0.7975066 | 0.467385 | 0.767135 | 0.43775 |
| *2016* | 0.816643 | 0.5248284 | 0.8207546 | 0.5367674 | 0.7997798 | 0.502454 | 0.746426 | 0.40810 |
| *2017* | 0.666244 | 0.2959695 | 0.6554913 | 0.2957195 | 0.6891878 | 0.310759 | 0.748317 | 0.38481 |
| *2018* | 0.754488 | 0.403504 | 0.747274 | 0.396092 | 0.7611583 | 0.382432 | 0.726082 | 0.35364 |
| *2019* | 0.679872 | 0.3198424 | 0.6766711 | 0.3128582 | 0.6779806 | 0.324812 | 0.697064 | 0.32817 |
| *2020* | 0.467719 | 0.1111111 | 0.4843354 | 0.1556317 | 0.4739346 | 0.147640 | 0.514269 | 0.15144 |
| *2021* | 0.76121 | 0.3956044 | 0.7403654 | 0.4675517 | 0.7857815 | 0.459098 | 0.824627 | 0.50935 |
| *2022* | 0.719017 | 0.338666 | 0.7330558 | 0.3544947 | 0.7258307 | 0.351580 | 0.686356 | 0.31220 |

**Table S2. Table of the percentage of the area of the habitat suitability and density maps where the value is more than 0.07 for the habitat suitability and more than 0.0075 for the density value. The red-shaded cells are the highest interannual values, while the blue-shaded cells are the lowest interannual values.**

| **Year** | **GAM_HS** | | **GAM_Dens** | | **Hurdle** | | **BRT** | |
| --- | --- | --- | --- | --- | --- | --- | --- | --- |
|  | **%** | **Area (km^2^)** | **%** | **Area (km^2^)** | **%** | **Area (km^2^)** | **%** | **Area (km^2^)** |
| *2008* | 10.00 | 65891.01 | 8.95 | 59336.17 | 6.86 | 45451.61 | 4.77 | 31454.20 |
| *2009* | 6.18 | 40636.87 | 5.15 | 34141.39 | 8.01 | 53224.33 | 3.07 | 20261.64 |
| *2010* | 12.79 | 84610.50 | 12.73 | 84708.50 | 8.46 | 56194.59 | 5.93 | 39219.24 |
| *2011* | 4.08 | 26730.29 | 3.88 | 25804.23 | 2.05 | 13838.89 | 1.87 | 12292.13 |
| *2012* | 4.40 | 28778.12 | 4.60 | 30540.72 | 2.34 | 15709.31 | 3.46 | 22660.35 |
| *2013* | 9.55 | 63082.31 | 10.09 | 67249.45 | 4.93 | 32918.59 | 5.46 | 36136.24 |
| *2014* | 7.11 | 46811.91 | 5.25 | 34888.02 | 3.76 | 25038.89 | 3.56 | 23481.36 |
| *2015* | 6.83 | 44987.80 | 6.19 | 41255.39 | 3.03 | 20393.70 | 5.14 | 34024.04 |
| *2016* | 10.05 | 66361.10 | 7.74 | 51341.86 | 4.94 | 32757.82 | 3.61 | 23825.09 |
| *2017* | 1.20 | 7833.08 | 1.04 | 7130.86 | 1.03 | 7031.78 | 1.42 | 9296.99 |
| *2018* | 14.16 | 94075.24 | 15.88 | 106564.09 | 7.30 | 48683.85 | 7.42 | 49321.86 |
| *2019* | 10.53 | 69499.44 | 9.32 | 61909.46 | 6.73 | 44702.88 | 6.35 | 41992.64 |
| *2020* | 1.94 | 12673.65 | 2.10 | 14037.29 | 1.44 | 9714.36 | 1.46 | 9563.80 |
| *2021* | 7.88 | 52277.08 | 5.18 | 35090.89 | 2.02 | 13827.65 | 5.60 | 37388.39 |
| *2022* | 8.94 | 58910.35 | 8.66 | 57830.07 | 3.44 | 22976.63 | 8.36 | 55524.88 |

**Table S3. Table of the percentage of the area of the habitat suitability and density maps where the value is more than 0.112 for the habitat suitability and more than 0.012 for the density value. The red-shaded cells are the highest interannual values, while the blue-shaded cells are the lowest interannual values.**

| **Year** | **GAM_HS** | | **GAM_Dens** | | **Hurdle** | | **BRT** | |
| --- | --- | --- | --- | --- | --- | --- | --- | --- |
|  | **%** | **Area (km^2^)** | **%** | **Area (km^2^)** | **%** | **Area (km^2^)** | **%** | **Area (km^2^)** |
| *2008* | 2.24 | 14643.55 | 1.18 | 7811.21 | 0.66 | 4456.41 | 0.49 | 3231.83 |
| *2009* | 0.00 | 15.69 | 0.09 | 642.04 | 0.92 | 6295.88 | 0.31 | 2009.11 |
| *2010* | 2.31 | 15086.95 | 0.98 | 6595.51 | 0.95 | 6474.89 | 0.92 | 6046.43 |
| *2011* | 0.00 | 0.00 | 0.17 | 1153.98 | 0.38 | 2606.32 | 0.20 | 1273.50 |
| *2012* | 0.05 | 327.70 | 0.26 | 1747.38 | 0.45 | 3084.80 | 0.57 | 3719.67 |
| *2013* | 0.00 | 0.00 | 0.79 | 5409.88 | 1.01 | 6878.57 | 0.84 | 5542.72 |
| *2014* | 0.00 | 0.00 | 0.20 | 1367.06 | 0.44 | 2985.14 | 0.26 | 1709.70 |
| *2015* | 0.00 | 0.00 | 0.94 | 6419.54 | 1.07 | 7294.41 | 0.84 | 5519.21 |
| *2016* | 0.05 | 333.01 | 0.01 | 49.24 | 0.09 | 592.01 | 0.41 | 2723.49 |
| *2017* | 0.00 | 0.00 | 0.00 | 0.00 | 0.00 | 32.94 | 0.11 | 707.67 |
| *2018* | 1.09 | 7123.58 | 0.84 | 5773.17 | 1.00 | 6862.88 | 1.57 | 10359.39 |
| *2019* | 1.39 | 9110.43 | 0.28 | 1928.61 | 0.57 | 3907.98 | 1.31 | 8655.68 |
| *2020* | 0.00 | 0.00 | 0.05 | 361.27 | 0.14 | 920.20 | 0.11 | 705.02 |
| *2021* | 0.00 | 0.00 | 0.24 | 1646.76 | 0.51 | 3459.77 | 0.93 | 6219.44 |
| *2022* | 0.27 | 1736.79 | 0.60 | 4105.48 | 0.84 | 5771.45 | 1.48 | 9784.25 |
